# Scalable X-ray scintillators with bright singlet-triplet hybrid self-trapping excitons

**DOI:** 10.1038/s41377-025-01869-z

**Published:** 2025-07-22

**Authors:** Shi-Yu Song, Chao-Jun Gao, Rui Zhou, Bing-Zhe Wang, Wen-Bo Zhao, Qing Cao, Yan-Wei Hu, Lin Dong, Kai-Kai Liu, Chong-Xin Shan

**Affiliations:** 1 Henan Key Laboratory of Diamond Optoelectronic Materials and Devices, Key Laboratory of Material Physics Ministry of Education, School of Physics and Key Laboratory of Zhongyuan Light, Zhengzhou University, Zhengzhou, China; 2 Joint Key Laboratory of the Ministry of Education, Institute of Applied Physics and Materials Engineering, University of Macau, Taipa, Macau, SAR 999078 China; 3 Institute of Quantum Materials and Physics Henan Academy of Sciences Zhengzhou, Zhengzhou, 450046 China

**Keywords:** Optical spectroscopy, Imaging and sensing

## Abstract

Size-scalable X-ray scintillators with high transparency and robust photon yield allow for imaging large objects with greater precision and detail. Solution-processable scintillators, typically crafted from quantum dots (QDs), are promising candidates for highly efficient scintillation applications. However, the restricted size and low transparency in QD-based scintillators lead to less efficient X-ray imaging for large objects requiring high resolution. Herein, we demonstrate a meter-scale ZnO QD scintillator with a visible range transmittance exceeding 96%, featuring bright singlet-triplet hybrid self-trapping excitons (STEs). The quantum yields (QYs) of singlet excitons and triplet excitons are 44.7% and 26.3%. Benefiting from a large Stokes shift and bright triplet excitons, the scintillator has a negligible self-absorption and elevated photon yields. Additionally, the scintillator exhibits exchange invariance, demonstrating identical optical performance upon exchanging the coordinates (*r*) of the QDs. Featuring bright singlet-triplet hybrid STEs and high transparency, the scintillator achieves high resolution X-ray imaging of 42-line pairs per millimeter (42 lp mm^−1^) at a meter scale. Moreover, demonstrations of 5000 cm^2^ X-ray imaging and real-time dynamic X-ray imaging are presented. The lowest detectable dose rate for X-ray detection is as low as 37.63 ± 0.4 nGy s^−1^. This work presents a novel sizable and transparent scintillator with bright singlet-triplet hybrid STEs, showcasing their potential in high-resolution and sizable object X-ray imaging.

## Abstract (web summary)

Size-scalable ZnO QD scintillators for advanced X-ray imaging.Introduction

## Graphical abstract

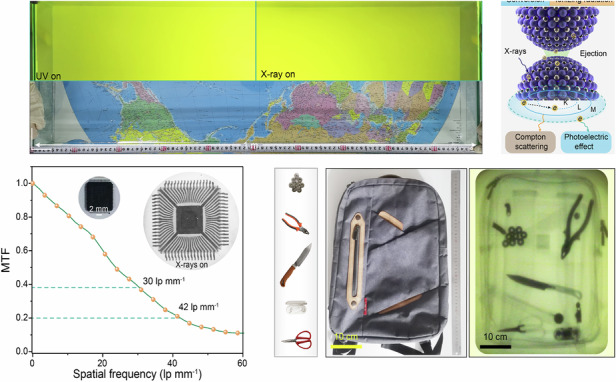

## Introduction

The interaction of X-ray photons with luminescent materials induces their emission of photons in the visible region, playing a key role in high-energy X-ray nondestructive detection and imaging^1–5^. Sizable scintillators with high transparency and robust photon yield enhance precision imaging of large objects, contributing to advancements in medical diagnostics^6–10^, industrial inspection^11–14^, and so on. The challenge lies in producing efficient large-area scintillators with high transparency, including traditional scintillators generated via the Czochralski method and emerging perovskite scintillators generated via chemical methods^15–18^. Small Stokes shift and strong self-absorption effect can greatly decrease the efficiency of light out-coupling of the perovskite scintillator^19–21^. The high toxicity and poor stability of lead components and heavy metal elements should be considered in practical applications. When X-ray photons bombard the inner-shell electrons of atoms of the luminescent materials, a tremendous number of high energy electrons through photon-electron interaction and secondary electrons through electron-electron scattering and Auger processes, are generated. The non-equilibrium electrons undergo relaxation before they can emit photons, resulting in electron–hole pairs^5^. The ionized electrons follow a 1:3 ratio in singlet and triplet, determined by spin-statistical charge recombination^22–24^. In most cases, conventional and perovskite scintillators only emit photons from singlet excitons after being irradiated because of the dark nature of triplet excitons^25,26^. Thus, when exposed to X-ray, the generated triplet excitons in these scintillators are useless and functionally irrelevant for scintillation.

Effectively utilizing triplet excitons presents an attractive way to enhance scintillation performance, especially in scintillators with fewer toxicant heavy metal elements. By chemically modifying the chromophores of thermally activated delayed fluorescence and phosphoresce molecules with halogen atoms, there has been a significant enhancement in X-ray absorption cross-section while preserving the bright nature of triplet excitons, leading to outstanding scintillation performance^27,28^. Despite advancements, reported organic scintillators exhibit poor transparency in the visible region, resulting in strong light scattering of the X-ray-induced photons. High transparency scintillators could achieve good spatial resolution in X-ray imaging^29–32^. Additionally, the complex molecular design and synthesis processes result in limited production capacity, limiting the application of these materials in large-area, high-resolution X-ray imaging. Solution-processable QD scintillators emerge as highly promising candidates for efficient scintillation applications, although it has proven challenging to synthesize a monodispersed population of ultrasmall quantum dots and to retain their solution-phase properties during assembly into scintillator solids. Therefore, the exploration of novel large-size and transparent scintillators, characterized by bright triplet excitons holds significant importance for low-dose, high-resolution, and large-area X-ray imaging.

In this study, a meter-scale scintillator with 96% transmittance in the visible region, is demonstrated through the disordered assembly of the ZnO QDs. Benefiting from the efficient utilization of bright singlet-triplet hybrid STEs in the scintillator, a photon yield of 16296 ± 1754 photons MeV^−1^ is achieved under X-ray excitation. Importantly, we have achieved the high resolution X-ray imaging of 42 line pairs per millimeter (42 lp mm^−1^) at a meter scale and the X-ray detectable dose rate of 37.63 ± 0.4 nGy s^−1^, surpassing most reported organic and conventional inorganic scintillators. Furthermore, we demonstrated high-resolution and large-area (5000 cm^2^) X-ray imaging of a backpack and broken pork rib through a single scan, and real-time X-ray imaging was also achieved.

## Results

**Fig. 1:**
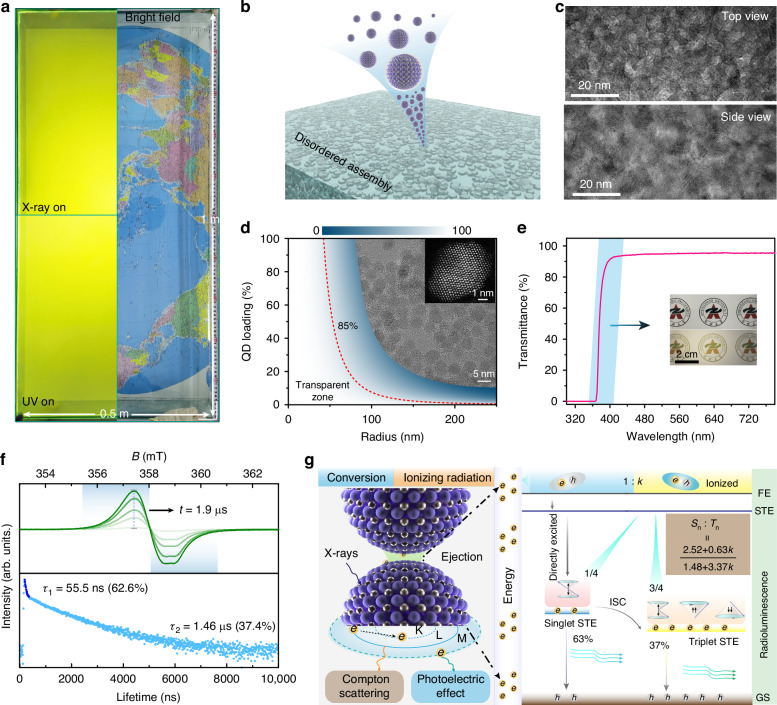
Illustration of design, structures, and mechanism of the scintillator. **a** Image of a meter-scale (1 m × 0.5 m) transparent scintillator through the disordered assembly of the ZnO QDs (left, UV and X-ray excitation; right, bright-field image). **b** Schematic representation of disordered assembly process of ZnO QDs scintillator. **c** The top-view and side view TEM images of the ZnO QD scintillator. **d** The simulated light transmittance spectrum of the scintillator with numerical parameter settings (*d* = 30 μm, *n*_*p*_ = 2.008, *n*_*m*_ = 1.6), the insert images are the TEM image and the high-resolution TEM image of the ZnO QDs. **e** Transmission spectrum of the scintillator. High transparency can be observed in the visible region, (insert: a 5 cm diameter ZnO QD scintillator of 300 μm in thickness). **f** Time-dependent ESR signal of ZnO QDs (top), PL decay curves of the ZnO QDs under 375 nm excitation (bottom). **g** Schematic diagram of the X-ray induced scintillation process in the ZnO QD scintillator

Solution-processable QDs with strong X-ray absorption ability and bright triplet excitons are ideal candidates for novel highly efficient X-ray scintillators^33,34^. Here, the meter-scale transparent scintillator was constructed using approximately ${10}^{21}$ ZnO QDs as building blocks through disordered assembly process (Fig. 1a). The manufactured scintillator mesaures 1 meter in length, 0.5 meter in width, and 0.5 mm in thickness, and its size is adjustable to suit various dimensions for practical applications through this method. The scintillator screen shows bright luminescence and radioluminescence (RL) when subjected to 365 nm UV light and X-ray source, and a world map positioned beneath the scintillator is clearly visible in daylight. The internal structure of the scintillator was investigated by transmission electron microscopy (TEM), and the QDs exhibited a disordered arranged state from the top and side view of the TEM images (Fig. 1b and Fig. 1c). The ZnO QDs exhibit a uniform size dispersion of 4.24 ± 0.20 nm from the TEM image, as shown in the inset of Fig. 1d and Figs. S1a and S1b. Notably, clear and integrated crystal structure can be observed from the high-resolution TEM image of QDs. In addition, dynamic light scattering spectrum was tested as shown in Fig. S1c, which confirmed the good dispersibility of ZnO QDs. The scintillator comprises an ensemble of the quasi-identical ZnO QDs, meaning that the QDs of the same type are fundamentally indistinguishable from each other. In the scintillator, there are n QD labeled with numbers r_1_, r_2_, …, r_n_, the optical performance (*O*) of the scintillator is defined as the function of space coordinate *r*. We then randomly exchanged the coordinates (*r*) of the QDs through redispersion, the scintillator displayed identical optical performance (*O*), including wavelength, intensity, and lifetime as shown in Fig. S2. Thus, the scintillator possesses exchange symmetry, namely luminescence exchange invariance. In this system, the interaction between the position and the ensemble optical properties can be described by the equations presented:

$${Pf}\left({r}_{1}{r}_{2}\ldots {r}_{n-1}{r}_{n}\right)=f\left({r}_{n}{r}_{2}\ldots {r}_{n-1}{r}_{1}\right)$$

$P$ is the exchange operator, specially, a chemical or physical reconstruction strategy for the scintillator. The exchange invariance of the scintillator indicates that the disordered assembly way is a universal method for the fabrication of the sizable scintillator with high transparency and good scintillation performance. ZnO QD with a wide band gap of 3.36 eV (Fig. S3), small diameter, and large Stokes shift (about 210 nm) is a reasonable candidate for the fabrication of transparent scintillators. The scattering cross-section of ZnO QDs in water, alcohol and SiO_2_ matrix was calculated based on Rayleigh scattering theory^35,36^, as shown in Fig. S4. The scattering cross section decreases from 4.42 × 10^−20^ m^−2^ in a water matrix to 2.68 × 10^−20^ m^−2^ in a silica matrix at the wavelength 500 nm. Such a difference was ascribed to the Rayleigh scattering, where a small refractive-index difference can significantly mitigate the scattering effect. The premix including ZnO QDs, ethanol, and water, presented a white solution with a low light transmittance. As the water and ethanol evaporated, the premix became gradually transparent and the pattern beneath the QDs composites gradually became clear (Fig. S5). In addition, the light transmittance (*T*) of a ZnO QD scintillator is shown in Fig. 1d (more calculation details see “Methods”). The simulated light transmittance spectra show that the transmittance is highly sensitive to the radius of QDs. The 90 wt % loading of 5 nm QDs exhibits comparable transparency to its 10 wt% counterpart. The volumetric fraction of ZnO QDs in the scintillator is about 95%, showcasing impressive transparency up to 96% in the wavelength range from 380 to 800 nm (Fig. 1e). It should be noted that each QD is isolated, and there are no distinct grain boundaries, resulting in a minimal difference in refractive index. The transparent nature and disordered inter-arrangement of the scintillator resemble glass, prompting us to rename it as a scintillator. The SEM image shows that the QD scintillator has a smooth surface in a large area (Fig. S6a), further decreasing the light scattering derived from the interface. The cross-section images revealed a dense packing of nanoparticles in the normal direction, and the film surface was considerably flat without any pinholes in a large area (Fig. S6b). Moreover, the surface roughness analysis of the ZnO QDs films from AFM results (upper pane, Fig. S7) reveals consistent morphology with a root−mean−square value of ∼6.1 nm, ensuring excellent uniformity. And the Intensity distribution (bottom pane, Fig. S7) demonstrates optical homogeneity. X-ray diffraction (XRD) pattern of ZnO QD scintillator and the XRD pattern of ZnO QD powders show the same diffraction peaks indexed to the ZnO (Fig. S8), indicating the isolation of ZnO QDs within the scintillator. In this disordered assembly process, discrete ZnO QDs were organized into spherical nanoparticles with different sizes (Fig. S9), resembling the snowball effect, while the QDs remained spatially well isolated (Fig. S10). In the end, the ZnO QDs underwent fusion to form a continuum structure, resulting in the formation of the ZnO QD scintillator. In the bottom of Fig. 1f, the nanosecond and microsecond lifetime of the ZnO QDs can be detected, and the lifetime of the ZnO QDs collected at 575 nm, can be well fitted by bi-exponential function with lifetimes of *τ*_1_ = 55.5 ns (63%) and *τ*_2_ = 1.46 μs (37%). The electron spin resonance (ESR) signal of ZnO QDs manifests a time-dependent nature, disappearing after excitation for 1.9 μs (top of Fig. 1f). This indicates the lifetime of triplet excitons is about 1.9 μs, which is in agreement with the long-lived component in luminescence lifetime. In addition to high transparency and large size, the scintillator possesses bright triplet excitons, which will be discussed in detail in the next part. The scintillation mechanism of the scintillator is illustrated in Fig. 1g. When exposed to X-ray photons, X-ray photons interact with the atom of the QDs by photoelectric effect and Compton scattering, inducing hot electrons generated from the directly excited process and ejected electrons from the ionized process. The hot electrons underwent thermal relaxation, forming excitons with holes, and returned to the ground state by emitting visible photons. The ejected electrons would be injected into the adjacent QDs, and the injected electrons would be populated in singlets and triplets following the ratio of 1:3, according to the rule of spin conservation. The ratio of directedly excited ZnO QDs to ionized ZnO QDs is set as 1: k, and the singlet-triplet states ratio (*S*_*n*_:*T*_*n*_) is (k + 4):(3k). For extremely ionizing radiation, k approaches infinity, the triplet states recombination would be favored. At the opposite *k* approaches zero, only singlet states are populated. For the ZnO QD scintillator, 37% of directly excited ZnO QDs, were populated into triplet state through intersystem crossing (ISC). Thus, the ultimate ratio of *S*_*n*_ to *T*_*n*_ is expressed as 0.63(1 + k/4): (0.37(1 + 1k/4) + 3k/4), which can be further simplified to (2.52 + 0.63k): (1.48 + 3.37k), when the scintillator exposed to X-ray photons. The bright nature of the triplet excitons increases the X-ray to visible photons conversion efficiency, by elevating the utilization rate of excitons.

The exciton nature of the ZnO QD scintillator and the dynamic processes were further investigated. Fig. S11a displays broadband PL at room temperature upon UV excitation, with a peak centered at around 575 nm. The broadband PL is a characteristic of STE emission, a feature previously confirmed in our earlier research^37^. This includes a large Stokes shift (210 nm) and a remarkable full width at half-maximum (FWHM) of 193 nm, due to strong coupling of the excitons with phonons. The absolute PL QY of the transparent ZnO QD scintillator after coating the silicon shell was measured (detail spectra can be found in Fig. S12), exhibiting an increase from 54.5% to 67.2%. This increase indicates that the high transparency is a significant factor contributing to the high light yields of ZnO QDs. The 3D contour plots of emission versus excitation spectra are presented in Fig. S13, clearly revealing a broad emission range and excitation-independent characteristics. The emission spectra, spanning from 350 to 850 nm, exhibit identical shapes and features across various excitation wavelengths, implying the recombination of a single initial excited state. Furthermore, the temperature-dependent PL spectra were recorded to investigate the electron-phonon coupling, STE binding energy, and Huang-Rhys factor (S), as shown in Fig. S11. The calculated STE binding energy is ∼212.6 ± 9.6 meV (Fig. S14) by the following equation:

$${I}_{T}={{I}_{0}}/\left(1+A\exp \left(-\frac{{E}_{b}}{{k}_{B}T}\right)\right)$$

Where *I*_*0*_ and *I*_*T*_ are the emission intensity at 0 K and T K, respectively; A is a constant, *E*_*b*_ is the binding energy, *k*_*B*_ is the Boltzmann constant. The FWHM of PL spectra undergoes broadening as the temperature rises from 77 K to 317 K (Fig. S11c). The Huang-Rhys factor (S) and optical phonon frequency *(ћω*_phonon_) can be described by the equation^38^:

$${FWHM}(T)=2.36\sqrt{{\rm{S}}}{{\hslash }}{\omega }_{{phonon}}\sqrt{\coth ({{\hslash }}{\omega }_{{phonon}}\,/2{k}_{B}\,T)}$$

**Fig. 2:**
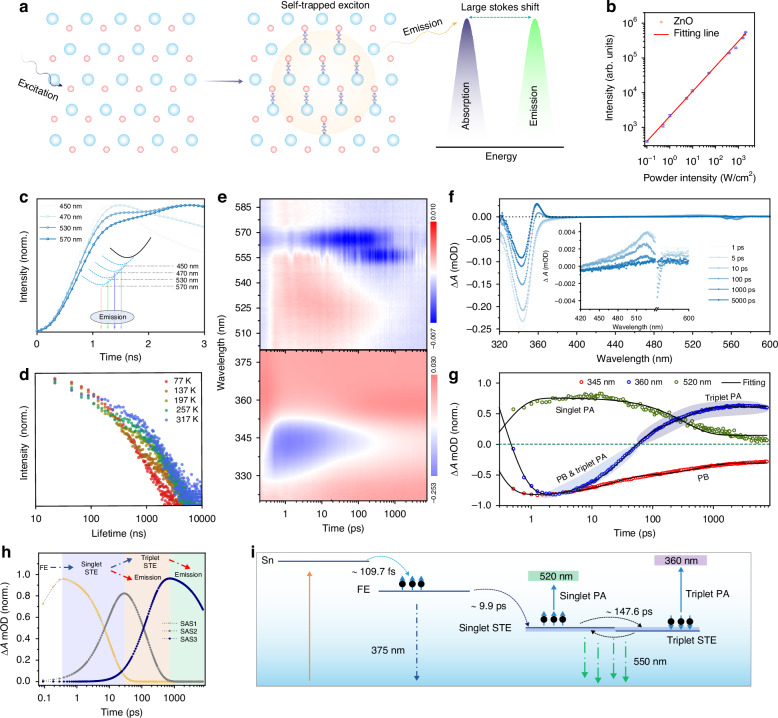
Investigation of luminescence mechanism for singlet-triplet hybrid excitons. **a** Schematic diagram of the generation of STEs in ZnO QDs upon photoexcitation. **b** The PL intensity signal as a function of the intensity of the laser power. *R*^2^_Adjusted_ = 0.996, for emission at 575 nm. **c** PL decay curves of the ZnO QDs at the selected wavelength from 450 nm to 570 nm. **d** Temperature-dependent PL lifetime decay spectra for the ZnO QDs. **e** TA contour maps of ZnO OQs between 20 fs to 7.5 ns under 280 nm excitation. **f** TA spectra collected at the indicated time from 1 to 5000 ps. **g** TA kinetic processes were probed at 345 nm, 360 nm, and 520 nm. The data were fitted using a biexponential decay model. **h** Species-associated difference spectra by the global analysis fitting. **i** Schematic diagram of the singlet-triplet hybrid STE emission process in the ZnO QDs

Where *ω*_*phonon*_ is phonon frequency, ℏ is reduced Planck constant, *T* is temperature, and *k*_*B*_ is Boltzmann constant. The calculated Huang-Rhys factor *S* and the phonon energy *ℏω*_*phonon*_ are 27.8 and 42.6 meV, respectively. This implies a strong electron-phonon coupling in ZnO QDs, due to the opposite symmetry vibration of zinc and oxygen atoms (as depicted in Fig. 2a), which facilitates the formation of STE with the large Stokes shift emission feature. The spectra from ZnO QDs versus excitation power over 4 orders of magnitude were recorded (Fig. S15), and the emission intensity shows a linear dependence on the excitation intensity (Fig. 2b), excluding the emission from permanent defects. The PL onset time demonstrates a wavelength-dependent characteristic, wherein photons with low energy show slower emergence compared with that of high energy, as shown in Fig. 2c. This phenomenon serves as direct evidence of STE emission. Additionally, in the 3D time-resolved emission spectra of ZnO QDs (Fig. S16), the presence of two distinct lifetimes is evident, signifying the existence of different exciton recombination processes. To explore the kinetics of carrier’s transfer, we further investigated the temperature-dependent PL decay dynamics properties from 77 K to 317 K, as shown in Fig. 2d and Fig. S17. The short lifetime (τ_1_) decreases from 212 ns to 63.5 ns with escalating temperature, while the long lifetime (*τ*_2_) gradually increases first (77–137 K) and then decreases (137–317 K). In the cryogenic region (77–137 K), the long lifetime is promoted compared with the high-temperature region (over 137 K), due to insufficient energy for electrons in the triplet state back to the singlet state^39,40^. The above results suggest that the emission of ZnO QDs originates from the singlet-triplet hybrid STE states. The average lifetimes (*τ*_ave_) of ZnO QDs at 180–300 K were recorded, as shown in Fig. S18, and the emission model can be fitted well with the functional relationship by the equation^41^:

$${\tau }_{{ave}}=\frac{3+\exp \left(-\frac{{\varDelta E}_{{ST}}}{{K}_{B}T}\right)}{3/{\tau }_{T}+1/{\tau }_{S}\exp \left(-\frac{{\varDelta E}_{{ST}}}{{K}_{B}T}\right)}$$

where *k*_*B*_ is the Boltzmann constant, *τ*_*S*_ and *τ*_*T*_ are the decay time of singlet and triplet states at 0 K (they are fitted decay lifetime at 77 K for this case), and *ΔE*_*ST*_ is the splitting energy gap between singlet and triplet, respectively. The fitted *ΔE*_*ST*_ is 48 meV, and such small singlet-triplet energy splitting was consistent with the theoretical calculations of 58 meV in our previous work^37^. To further investigate the dynamic processes of the generated carriers after excitation, excited state spectra of ZnO QDs were performed using broad femtosecond transient absorption (TA) spectroscopy in Fig. 2e. Negative Δ*A* features (blue) are discernible in the spectral region spanning regions of 330–350 nm and 560–570 nm, while positive features (highlighted in red) are evident within the wavelength range of 490–540 nm. The TA spectra at different time delays, along with the corresponding decay spectra at 345, 360, and 520 nm, are presented in Fig. 2f, g. The 330–350 nm spectral region is dominated by ground-state photobleaching (PB), exhibiting a consistent match with the steady-state absorption spectrum. Noting that, the signal at around 360 nm shows a negative response within 100 ps, followed by a transition to a positive signal thereafter. This alteration serves as a direct indicator of triplet photoabsorption (PA). Thus, the decay curve at 360 nm represents a hybrid signal including both PB and triplet PA. The broad excited-state absorption in the green region from 485 to 520 nm corresponds to the singlet state PA, and the corresponding lifetime associated with the state at 520 nm state is ~1 ns. Species-associated difference spectra (SAS) of the ZnO QDs TA data reveal three components with lifetimes of 9.9 ps, 147.6 ps, and 18.7 ns as shown in Fig. 2h and Fig. S19. The kinetics of carriers’ evolution in various excited energy levels can be clearly observed. The generated hot carriers undergo relaxation to the free excitons (FE) state within 109.7 fs, followed by a further relaxation to the singlet STE state within 9.9 ps due to the strong electron-phonon coupling. The singlet STE state evolution can be directly resolved by the decay curves at 520 nm. Subsequently, the singlet STE state converts into the long-lived species within 147.6 ps through an intersystem crossing (ISC) process, characterized by distinct excited-state absorption at 360 nm. Compared with PL decay spectra, the average lifetimes of ZnO QDs at 375 nm and 575 nm are 2.7 ns and 1030 ns, and a plausible mechanism model for carrier evolution is shown in Fig. 2i. When ZnO QDs are exposed to X-ray photons, hot electrons are generated from the direct excitation, and ejected electrons are generated from the ionization process. Subsequently, the generated hot electrons then undergo thermalization and produce low-energy excitons. Next, some electrons transition from the singlet state to triplet states through the ISC process, which can efficiently populate triplet excitons. The electrons in excited singlet and triplet states return to the ground state by emitting visible photon. The above results confirm that the emission of the ZnO QDs is attributed to the radiative recombination of singlet-triplet hybrid STE, rendering it highly advantageous for scintillator applications.

**Fig. 3:**
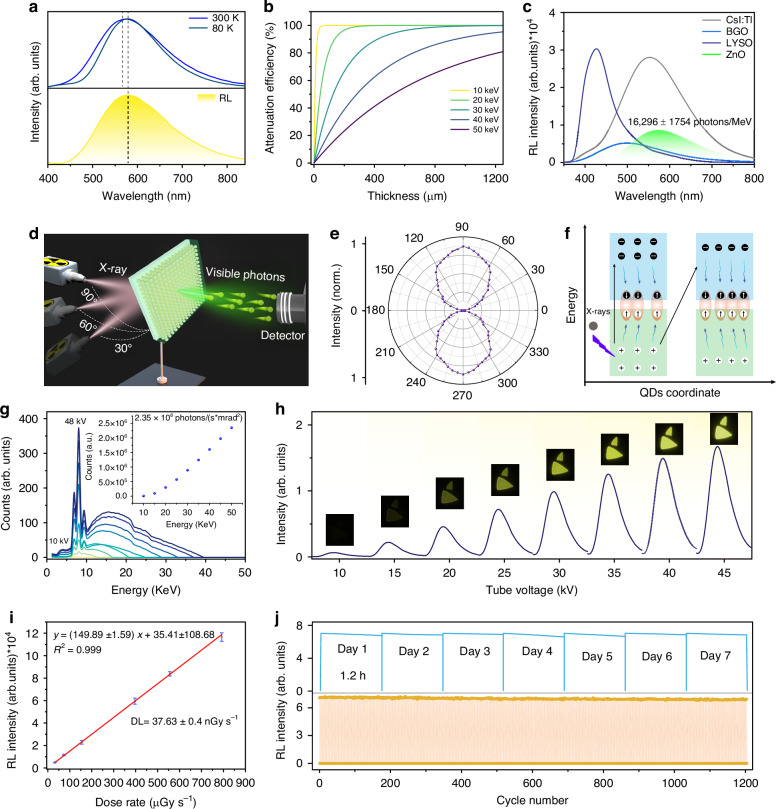
Investigation of singlet-triplet hybrid STEs emission upon X-ray excitation. **a** Normalized PL at 80 K and 300 K, and RL spectra of the ZnO QD scintillator. **b** X-ray attenuation efficiencies of ZnO QD scintillator versus varied thicknesses, from 10 keV to 50 keV. **c** Normalized RL spectra of the ZnO QD scintillator, CsI: Tl and Bi_4_Ge_3_O_12_ (BGO). **d** Schematic diagram showing omnidirectional radiation detection. **e** The light intensity in different directions (0-360 degrees) at the same dose rate. **f** Illustration of the mechanism in the formation of multiple excited charge carriers in ZnO QDs. **g** X-ray photon energy spectra generated at accelerating voltage from 10 kV to 45 kV. **h** RL spectra of ZnO QDs under different X-ray photons, the insets are the corresponding images. **i** The dose rate dependence of the RL intensity of ZnO QDs. The detection limit of 37.63 ± 0.4 nGy s^−1^ is derived from the slope of the fitting line, with a signal-to-noise ratio of 3. **j** The emission photostability at a peak of 575 nm for the ZnO QDs versus continuous irradiation (top) and repeated on–off cycles of X-rays (bottom)

The RL spectra of the ZnO QD scintillator under excitation of a 45 kV X-ray source and UV excitation (at 80 and 300 K) were recorded, as shown in Fig. 3a. A broad and intense yellow RL band, peaking at 590 nm can be observed, suggesting the RL originates from the emission of hybrid excitons. The RL spectrum, resembling that of ZnO QDs measured at 80 K but with a 15 nm red-shift in peak position compared to the PL spectrum measured at 300 K, implies the pivotal involvement of triplet excitons in the ZnO QD scintillator. Moreover, the temperature-dependent PL spectra were further recorded and only the intensity of PL spectra decrease was observed as the temperature from rises 298 K to 418 K due to thermal effect (Fig. S20). Thus, the 15 nm red shift in the self-trap emission profile when heating the NPs from 80 K to 300 K is due to the contribution of triplet excitons. Fig. 3b and Fig. S21 illustrate the X-ray photon absorption coefficient of ZnO QD scintillator (highest atomic number *Z*_max_ = 30, Kα = 8.6 keV) as a function of thickness at an X-ray photon energy of 10–50 keV^42^. It can be found that the ZnO QD scintillator can absorb 90% of the X-ray photon energy at a thickness of 500 μm. Thus, it is reasonable to expect that the hybrid singlet-triplets of the scintillator can be populated under the excitation of photons with low energy ranging from a few hundred eV to dozens keV, particularly with a thickness over 500 μm. Besides, the high PL QY, transparency, zero self-absorption, and intense light output of the ZnO QD scintillator can lead to a high scintillation light yield. The X-ray to light conversion efficiencies were measured, as shown in Fig. 3c and Fig. S22. The ZnO QD scintillator exhibits light conversion efficiencies of 16296 ± 1754 photons MeV^−1^, presenting a more favorable performance compared to the widely used commercial bulk BGO scintillator^43,44^. Furthermore, the ZnO QD scintillator screen was assessed for its ability to absorb X-ray photons from all angles, as illustrated in Fig. 3d. Tuning the X-ray source under a 360-degree circular motion to excite the scintillator, the RL intensity was measured with an optical fiber spectrometer, as shown in Fig. 3e. The RL intensity shows a degree-dependent characteristic property, laying a solid foundation for the design of X-ray imaging applications. In addition, a comparative experiment with different detection angles approach shown in Fig. S23 was designed. The intensity of the RL gradually increases as the irradiation angles increase from 0° to 90°, primarily attributed to the optical channel crosstalk, causing the reduction in the transmitted efficiency of the RL. Fig. 3f schematically shows the internal mechanism of the RL in ZnO QD scintillators. Upon X-ray irradiation, inner electrons are ejected from the atom and substantial hot electrons and holes can be created. Following X-ray irradiation, the ejected electrons subsequently inject into nearby QDs following a singlet-triplet ratio of 1:3. The remaining hot electrons then undergo relaxation through electron-photon interaction, following a singlet-triplet ratio of 0.63:0.37. The energy distribution spectra of the continuous X-ray sources, containing the characteristic X-ray peak and the bremsstrahlung, are shown in Fig. 3g. The X-ray flux can be as high as 10^6^ photons (s*mrad^2^)^−1^, and the average photon energy increases from 6.85 keV to 16.03 keV as the operating voltage increases from 10 kV to 45 kV. In Fig. 3h, the RL intensity of the ZnO QD scintillator shows a significant enhancement with increasing voltage, indicating an effective X-ray photons conversion to visible photons. The integrated RL intensity of the ZnO QD scintillator shown in Fig. 3i presents an excellent linear response to the X-ray dose rates and a low detection limit of 37.63 ± 0.4 nGy s^−1^ for this transparent ZnO QD scintillator can be derived from the fitting curve when the signal-to-noise ratio is 3, which is lower than the typical medical imaging dose (5.5 µGy s^−1^)^45–47^. The RL intensity of the ZnO QD scintillator can remain constant after working for 40 days (Fig. S24). The luminescence intensity of the ZnO QD scintillator remains consistent with increasing relative humidity from 50% to 90%, indicating that the luminescence of ZnO QD scintillator was not affected even at a high relative humidity (Fig. S25). The photostability was further examined under continuous or repeated cycles of X-ray illumination, and it can be activated repeatedly with no obvious decrease, indicating the good photostability of the ZnO QD scintillator as shown in Fig. 3j. In addition, Fig. S26 shows the cost analysis of the ZnO QD scintillator compared to other widely reported scintillators. The lower cost and ease of preparation of the ZnO QD scintillator may arouse intense interest in its commercial competitiveness in view of the scintillator application.

**Fig. 4:**
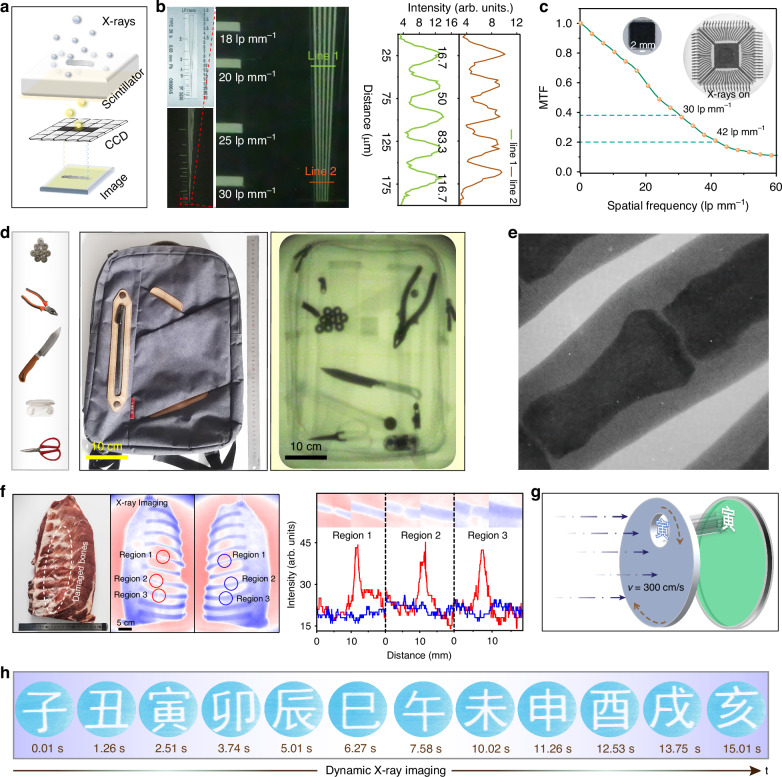
High-resolution and large-area X-ray imaging utilizing ZnO QD scintillator. **a** Schematic diagram of the experimental setup used for X-ray imaging of samples. **b** Photograph of the standard X-ray test-pattern plate. X-ray images of the test-pattern plate based on ZnO QD scintillator (X-ray tube voltage: 40 kV, dose rate: 27.1 μGy s^−1^). **c** Spatial resolution of the X-ray imaging system, characterized by the modulation transfer function. The spatial resolution of the ZnO QD scintillator is 30 lp mm^−1^ at MTF = 0.4 and 42 lp mm^−1^ at MTF = 0.2. Insert: photographs of the test chip and the corresponding X-ray image. (dose rate: 16.9 μGy s^−1^) **d** Bright-field (left)- (left) and dark-field (right) photographs of the backpack, recorded before and after X-ray exposure (dose rate: 7.1 μGy s^−1^). **e** X-ray image of the finger (dose rate: 4.1 μGy s^−1^). **f** X-ray images of broken pork rib using the large area ZnO QD scintillator (dose rate: 15.0 μGy s^−1^). **g** Schematic diagram of the experimental setup used for real-time X-ray imaging. **h** Dynamic X-ray images of twelve patterns

Regarding the excellent RL performance, highly transparence, and large size of the ZnO QD scintillator, we next implemented X-ray imaging with the ZnO QD scintillator as an imaging screen, and the corresponding imaging setup is illustrated in Fig. 4a. The RL intensity exhibits a notable increase with the growing thickness of the ZnO QD scintillator, while the transmittance remains 90% at 1 mm. The spatial The spatial resolution of the ZnO QD scintillator with 0.1 mm thickness for for X-ray imaging was assessed by using an X-ray standard test pattern panel consisting of lines with widths ranging from 1 to 30 lp mm^−1^. The X-ray imaging of the linear mask closely mirrored the corresponding optical image, as revealed on the ZnO QD scintillator screen (left of Fig. 4b). Lines with a width of 16 µm can be clearly resolved (right of Fig. 4b), demonstrating a spatial resolution of 30 lp mm^-1^. As the film thickness increases to 1 mm, the imaging resolution decreases to 11.8 lp mm^−1^ (Fig. S27) due to the waveguiding and light scattering effects. Note that the modulation transfer function (MTF) provides a spatial resolution of 42 lp mm^−1^ at the value of 0.2 (Fig. 4c), and the clear internal structure of a chip can be observed, as shown in the inset of Fig. 4c. The spatial resolution is much higher than the recently reported scintillator screens, such as CsPbBr_3_ (12.5 lp mm^−1^)^48^, Cs_3_Cu_2_I_5_ (6.8 lp mm^−1^)^49^, lanthanide-doped nanoscintillators (25 lp mm^−1^)^50^ and so on. This is attributed to the superior transparency and excellent RL performance of the ZnO QD scintillator. Subsequently, we evaluated the feasibility of sizable ZnO QD scintillator screen (3600 cm^2^) for X-ray imaging in safety inspection. The X-ray images of a backpack with various samples (bearings, pliers, knife, earphones, scissor) were monitored, as shown in Fig. 4d. The outline of the internal objects can be distinctly observed, and the color variations were a result of their differences in X-ray absorptions. The internal structures of a small computer mouse can be observed using the sizable scintillator (Fig. S28a), indicating the ability for objects of various sizes. The ZnO QD scintillator can also be employed for imaging the internal structure of a finger (Fig. 4e) and a moving finger (Fig. S28b). Obvious biological tissue phase contrast and clear joint details can be observed in the randomly selected X-ray images at different time points. Moreover, the feasibility of large-area medical single-scan X-ray imaging was explored, the optical image and pseudo-color images are shown in Fig. 4f. The original X-ray images are shown in Fig. S28c, the internal biological tissue and bones of the pork ribs were observed under high X-ray photon energy. Subsequently, the details of the broken bone joint were analyzed, and obvious increases in gray value in the fractured area can be observed, as further revealed by the magnified images in the inset. In our case, large-area X-ray imaging can cover an expansive area of 2500 cm^2^ through a single scan, representing a record for a single large-area X-ray imaging scan to date to the best of our knowledge. In addition, real-time X-ray dynamic imaging (24 frames per second) was demonstrated, and the corresponding sketch map is illustrated in Fig. 4g. The twelve Chinese zodiac characters exhibit clear identification of sharp edges without the ghosting effect in the captured photos at different time intervals, attributed to the fast response of the ZnO QD scintillator (Fig. 4h). The sizable ZnO QD scintillator, exhibits efficient conversion of X-ray photons into visible light, characterized by high transparency, notable RL performance, and superior spatial resolution, rendering it suitable for diverse applications in X-ray imaging and safety inspection at a low cost.

## Discussion

In conclusion, the sizable ZnO QD scintillator featuring singlet-triplet hybrid STEs was demonstrated through a disordered assembly technique, with the aim of achieving high-resolution X-ray imaging for large objects. Compared to the way of QDs coupled with the organic polymer and conventional Czochralski method, the key attributes of the ZnO QD scintillator are room temperature solution-based large-area synthesis, high QD loading, relatively low toxicity, high transparency, and robust RL. Importantly, our work has efficiently harnessed the X-ray-induced generation of hybrid STE, not only showcasing the potential for enhanced RL performance but also laying the foundation for the design of innovative scintillators.

Moreover, the successful creation of a large transparent ZnO QD scintillator, measuring 1 m × 0.5 m and with 96% transparency, represents a significant stride forward. This scintillator enables high-resolution (42 lp mm^-1^), low-dose (37.63 ± 0.4 nGy s^−1^), large-area (5000 cm^2^), and real-time X-ray imaging. This scintillator route may help redefine the paradigm for QD-based scintillator manufacturing, which not only provides a versatile approach for the preparation of QD scintillators but also may open a series of exciting applications for advancing radiation detection and medical imaging^51–53^.

## Materials and methods

### Materials

All the chemicals were purchased from Aladdin Chemistry Co. Ltd. (Shanghai, China). and they were all analytical grade: Zinc acetate dihydrate (Zn (Ac)_2_ ∙ 2H_2_O), potassium hydroxide (KOH), (3-aminopropyl) triethoxysilane (APTES) were purchased from Aladdin Chemistry Co. Ltd. (Shanghai, China). The source of absolute ethanol was Tianjin Kaitong Chemical Reagent Co. Ltd. (Tianjin, China).

### Synthesis of the ZnO QDs

In the first step, 923.7 g Zn(Ac)_2_ ∙ 2H_2_O and 343.3 g KOH were dispersed in 20 L and 5 L of pure ethanol. Subsequently, the KOH solution was added into the Zn(Ac)_2_ ∙ 2H_2_O solution followed by continuous stirring for 2 h. The complex solution above was filled with 66.5 mL APTES and stirred continuously for 8 h. The precipitate was centrifuged and washed with absolute ethanol three times. Eventually, ZnO QDs were obtained after drying in a blast oven at 70 °C for 12 h.

### Growth of the ZnO QD scintillator

In a typical procedure, the ZnO QDs are dispersed in water (1:2 by mass) and sonicated for 30 min to ensure its homogeneity. The resulting composites were degassed in a vacuum container to remove air bubbles. The viscous mixture solution was carefully coated on the quartz plates (or poured into a round plastic plate) to fabricate the scintillator. Then the coated plates were finally heated at 45 °C for 6 h. After cooling to room temperature, and the sample was left for 2 days or even longer to allow the solution to fully evaporate. The as-fabricated scintillator was then used for X-ray imaging.

### The light transmittance (T) of the ZnO QD scintillator calculations

Rayleigh cross section was calculated following the equation:

$$\sigma =\left(\frac{32{\pi }^{5}N{r}^{6}}{{\lambda }^{4}}\right)$$

where *N*, *r* and *λ* represent a refractive-index-related constant, the radius of QDs and the wavelength of incident light, respectively. Then, the Rayleigh cross-section values of the QDs dispersed in water, ethanol, and silica were calculated by setting values to parameters, where the QDs radius was ~5 nm. The *N* value was calculated following the equation as below:

$$N={n}_{p}^{4}{\left[\frac{{(\frac{{n}_{m}}{{n}_{p}})}^{2}-1}{{(\frac{{n}_{m}}{{n}_{p}})}^{2}+2}\right]}^{2}$$

Where ${n}_{m}$ and ${n}_{p}$ represent the refractive-index-related constant of matrix and QDs. According to Rayleigh scattering theory, the light transmittance (T) of a particle containing scintillator is described by the formula:

$$T=\exp \left(\frac{-32{\pi }^{4}N{V}_{f}{\rm{d}}{r}^{3}}{{\lambda }^{4}}\right)$$

where *N, V*_*f*_*, d, r*, and *λ* represent a refractive-index-related constant, QDs volumetric fraction, thickness of the composite scintillator, QDs radius, and the light wavelength.

### Structural characterization

Transmission Electron Microscope (TEM) images were measured on TEM (TecnaiG2 F20 S-TWIN) at 200-kV acceleration voltage to characterize the microstructures of all the ZnO QDs. FTIR spectra were recognized on an FTIR (Nicolet 6700) spectrometer. XRD patterns were collected with a Smartlab X-ray diffractometer (Rigaku, Japan) using Cu Kα radiation. XPS patterns were obtained using an X-ray photoelectron spectrometer to measure the bonding state of the QDs. The cross-section and surface of the ZnO QD scintillator were observed by a JSM-6700F (JEOL, Japan) field emission scanning electron microscope (FESEM). Special Aberration Corrected Transmission Electron Microscope (AC-TEM) images of the ZnO QD scintillator were measured by TEM (FEI Talos F200) under an acceleration voltage of 200 kV. Photographs of X-ray-induced luminescence and RL-based X-ray imaging were acquired with a digital camera (Canon EOS 5D Mark IV) in an all-manual mode.

### Optical characterization

The fluorescence spectra and excitation-emission spectra of the ZnO QDs were measured on an Edinburgh FLS1000 fluorescence spectrophotometer. Ultraviolet-visible (UV–vis) absorption and transmittance spectra of the ZnO QDs were obtained using Hitachi UH 4150 UV–vis spectrophotometers, respectively. The lifetimes, kinetic measurements, and time-resolved emission spectra were measured using an Edinburgh FLS1000 fluorescence spectrophotometer equipped with a microsecond flash-lamp (uF2), a xenon arc lamp (Xe2), and a nanosecond hydrogen flashlamp (nF920), respectively. The temperature-dependent spectra of the samples were measured using an FLS1000 spectrophotometer equipped with temperature control instruments (Advanced Research Systems) at various temperatures from 317 to 12 K. Absolute photoluminescence quantum-yield measurements at room temperature were performed on an FLS1000 spectrometer with calibrated integrating sphere. For the transient absorption spectrum measurements, a regeneratively amplified femtosecond Ti: Sapphire laser system (150 fs, 1 kHz) was employed. In this system, a 280 nm pump pulse was focused on the samples through an automated TOPAS Optical Parametric Amplifier (OPA). Then, the probe beam was focused and passed through the sample by an Al parabolic reflector. After passing through the sample, the transmission changes of the probe light were detected by a fiber spectrometer. And the absorbance change of the sample was calculated with two adjacent probe pulses. 50 mg mL^−1^ ZnO QDs solution was selected to measure the transient absorption spectrum.

### Radioluminescence measurement for the ZnO QD scintillator

The measurement of X-ray-induced luminescence was performed using a solid ZnO QD scintillator. The RL spectra were recorded using an Edinburgh FLS-1000 fluorescence spectrophotometer with a miniature X-ray source. The 360-degree omnidirectional radiation detection RL spectra were measured through our home-built X-ray imaging system including the rotating test platform with an optical fiber spectrometer.

### High resolution X-ray imaging

The diameter 3.5 cm and thickness of about 0.1 mm ZnO QD scintillator film was employed to measure the imaging properties in our home-built X-ray imaging system. The micro-focus X-rays source (focus size of 35 μm, accelerating voltage of 10–50 kV) was supplied by the Wisman Co. Ltd. (Xi’an, China). And the micro-focus X-ray source is much smaller than the commonly used X-ray sources with the focus size of 0.1 mm. The X-ray images of objects are reflected by the macro lens (Canon EF 100 mm f/2.8 USM Macro) coupled with rectangular reflecting prism and then recorded by a Canon EOS 5D Mark IV camera (Pixel size: 6.5 μm). The imaging system is in a reflection mode, which can effectively avoid the noise signal generated in the complementary metal oxide semiconductor sensor of camera caused by extra X-ray photons, without sacrificing the spatial resolution. During the imaging test, the X-ray voltage was selected to be 40 kV, the camera exposure time was 3 min, the ISO was 800, and the DOF was 5.0.

### Large-area X-ray imaging and dynamic X-ray imaging

The large area X-ray imaging was performed by our home-built large area X-ray imaging system, which include a large area X-ray source (focus size: 0.8 mm), large area ZnO QD scintillator film (70 cm × 70 cm). The ZnO QDs were dissolved in water, forming the solution with a concentration of 0.1 mg mL^−1^–2 g mL^−1^ drop casting on glass, quartz or polyvinylchloride (PVC) substrates and dried naturally in air. The thickness and the area of films were controlled via changing the solution concentration and the times of drop-casting. A 0.2 mm thick PVC film fixed on a stainless-steel frame is used as the substrate to support the large area ZnO QD scintillator film for imaging during large area measurements. The large area X-ray scintillation detector was constructed by preparing of meter-scale ZnO QD scintillator with a layer thickness of 800 μm. In order to maximize the collection of visible photons, the ZnO QD scintillator was coupled with a normal digital camera. For X-ray imaging, a backpack filled with different samples (bearings, pliers, knife, earphones, scissor) and a broken pork rib were utilized. Dynamic X-ray imaging was achieved using mechanical Chinese character turntables as imaging objects that were equipped with a scintillator. The objects and the ZnO QD scintillator wafers were arranged vertically before the incident X-rays, with the scintillators anchored directly behind them. The object’s speed of rotation is 300 cm s^−1^ and its record rate is 99 frames per second.

## Supplementary information

Support informarion

## Data Availability

All relevant data are included in this article and its Supplementary Information files. All data underlying this study are available from the corresponding author K.-K.L. upon request.
