# Supplementary material for: Scalable X-ray scintillators with bright singlet-triplet hybrid self-trapping excitons
Source: Light Sci Appl. 2025 Jul 22;14:249. doi: 10.1038/s41377-025-01869-z (PMC12283921; doi:10.1038/s41377-025-01869-z)
Supplement: Supplementary file 1 — Support informarion [file 41377_2025_1869_MOESM1_ESM.docx]

**Supplementary Materials**

**Scalable X-Ray Scintillators with Bright Singlet-Triplet Hybrid Self-Trapping Excitons**

Shi-Yu Song^1,#^, Chao-Jun Gao^1,#^, Rui Zhou^1^, Bing-Zhe Wang^2^,Wen-Bo Zhao^1^, Qing Cao^1^, Yan-Wei Hu^1^, Lin Dong^1^, Kai-Kai Liu^1,3,✉^ and Chong-Xin Shan^1,✉^

*^1^Henan Key Laboratory of Diamond Optoelectronic Materials and Devices, Key Laboratory of Material Physics Ministry of Education, School of Physics and Key Laboratory of Zhongyuan Light, Zhengzhou University, Zhengzhou, China*

*^2^* *Joint Key Laboratory of the Ministry of Education, Institute of Applied Physics and Materials Engineering, University of Macau, Taipa, Macau SAR 999078, China*

*^3^Institute of Quantum Materials and Physics Henan Academy of Sciences Zhengzhou 450046, China*

*^#^These authors contributed equally to this work.*

*^✉^e-mail:* [*liukaikai@zzu.edu.cn*](mailto:liukaikai@zzu.edu.cn)*;* [*cxshan@zzu.edu.cn*](mailto:cxshan@zzu.edu.cn)*.*

**Contents**

[Figure S1. ZnO QDs' size distribution. 3](#_Toc179400927)

[Figure S2. The optical performance of the ZnO QD scintillator. 4](#_Toc179400928)

[Figure S3. Absorption spectrum for the ZnO QDs 5](#_Toc179400929)

[Figure S4. The theoretical calculation of Rayleigh scattering cross-section (σ) for the ZnO QDs at various wavelengths in water, ethanol, and SiO_2_.. 6](#_Toc179400930)

[Figure S5. Photographs of the scintillator from opaque-to-transparent (left), and the schematic diagram (right) of the varied scattering effect from two interfaces: EtOH/QDs and SiO_2_/ QDs. 7](#_Toc179400931)

[Figure S6. The surface of the ZnO QD scintillator captured in the SEM image 8](#_Toc179400932)

[Figure S7. AFM image of the ZnO QD scintillator films 9](#_Toc179400933)

[Figure S8. XRD pattern of the ZnO QD powders and ZnO QD scintillator films 10](#_Toc179400934)

[Figure S9. SEM images of the ZnO QD scintillator captured at various formation processes. 11](#_Toc179400935)

[Figure S10. TEM image of the ZnO QD vitreous scintillator. 12](#_Toc179400936)

[Figure S11. Absorbance, PL, and PL excitation spectra of ZnO QDs. Temperature-dependent PL spectra and FWHM emission of ZnO QDs. 13](#_Toc179400937)

[Figure S12. The PL QY of ZnO QDs tested for five times before and after coating the silicon shell. 14](#_Toc179400938)

[Figure S13. Excitation-Emission Characteristics of the ZnO QD scintillator 15](#_Toc179400939)

[Figure S14. Binding energy fitting results of the ZnO QDs 16](#_Toc179400940)

[Figure S15. The PL spectra of the ZnO QDs under excitation of laser with varying powers 17](#_Toc179400941)

[Figure S16. 3D time-resolved emission spectra and the PL lifetime changes of the ZnO QDs 18](#_Toc179400942)

[Figure S17. The analysis results of temperature-dependent PL lifetime for the ZnO QDs 19](#_Toc179400943)

[Figure S18. Temperature-dependent average lifetimes of the ZnO QDs. 20](#_Toc179400944)

[Figure S19. Global analysis TA spectra of the ZnO QDs 21](#_Toc179400945)

[Figure S20. Temperature-dependent PL spectra of ZnO QDs measured from 298 to 418 K. 22](#_Toc179400946)

[Figure S21. Calculated X-ray attenuation efficiencies for BGO, CsI:Tl, and ZnO with varying thicknesses. Also, absorption spectra of ZnO, Zn, Si, and O concerning X-ray energy. 23](#_Toc179400947)

[Figure S22. The light yield test system 24](#_Toc179400948)

[Figure S23. Simulated light intensity and measured light intensity in different directions at the same X-ray dose rate. 26](#_Toc179400949)

[Figure S24. RL stability of the ZnO QD vitreous scintillator 27](#_Toc179400950)

[Figure S25. Humidity stability of the ZnO QD vitreous scintillator 27](#_Toc179400950)

[Figure S26. Cost/price comparison of the ZnO QD and other scintillators. 28](#_Toc179400951)

[Figure S27. The spatial resolution of the ZnO QD scintillator with different thickness 29](#_Toc179400952)

[Figure S28. Digital photograph and corresponding X-ray images using the ZnO QD scintillator 30](#_Toc179400953)


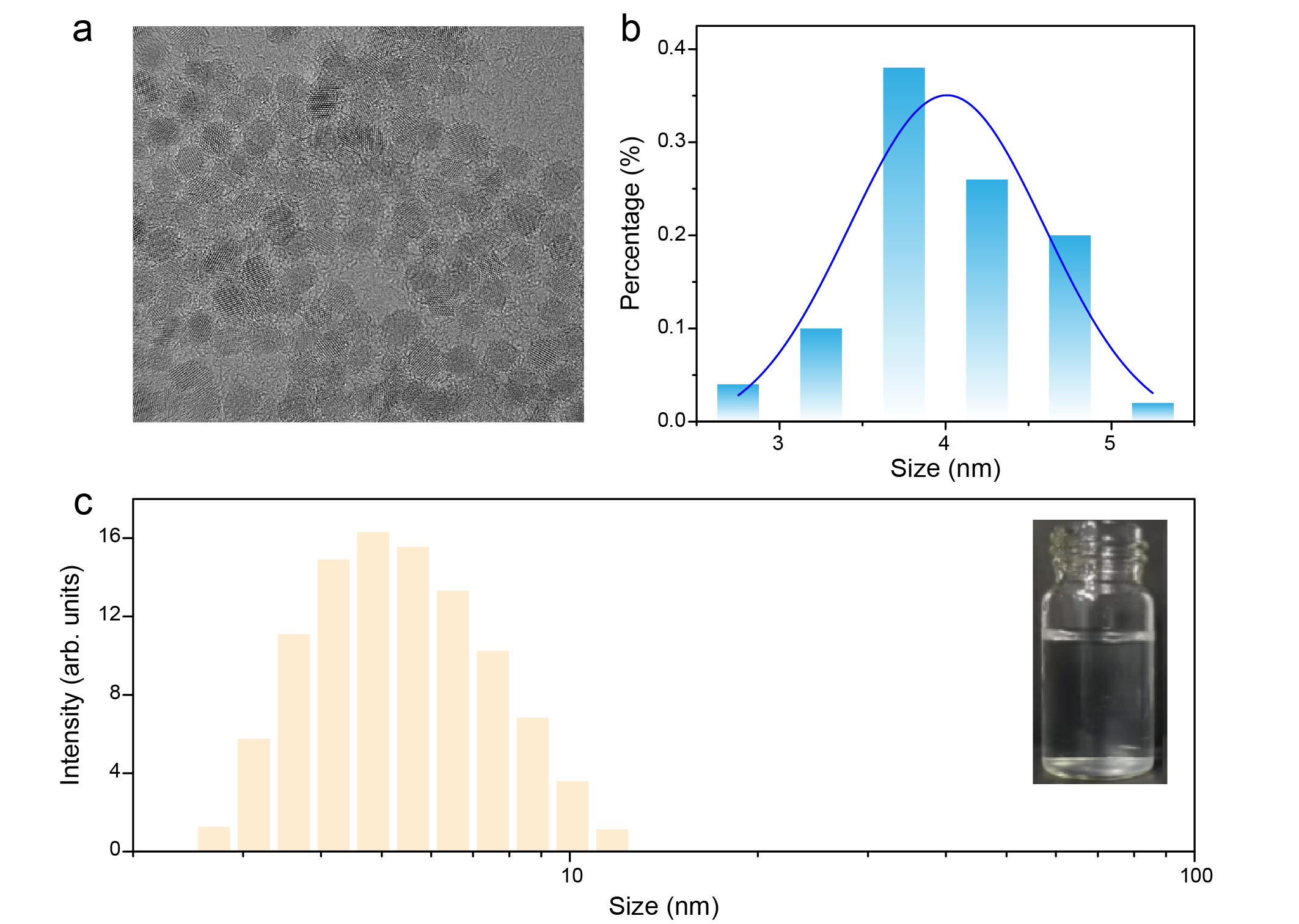


# Figure S1. (a) TEM image and (b) the size distribution of the ZnO QDs. (c) The size distribution of the ZnO QDs monitored by dynamic light scattering.


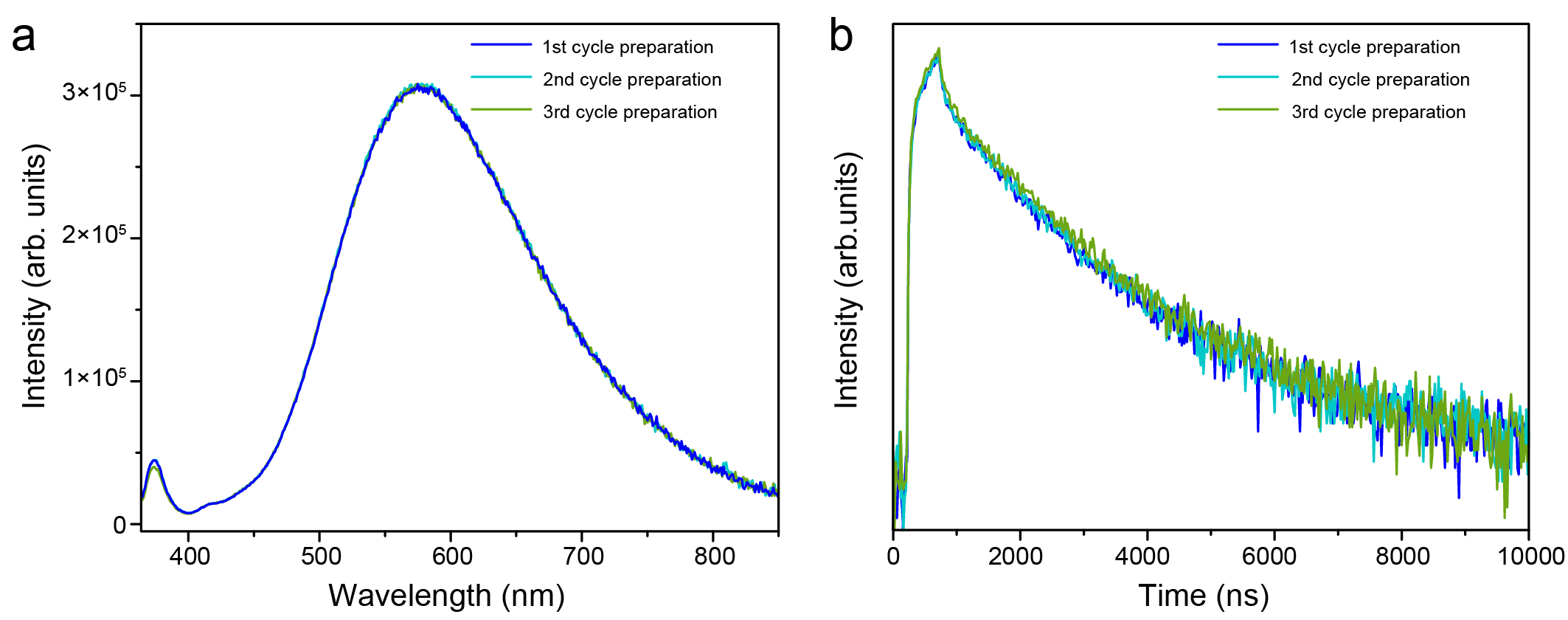


# Figure S2. (a) PL and (b) PL lifetime decay spectra of the ZnO QD scintillator after multiple cycles of preparation.


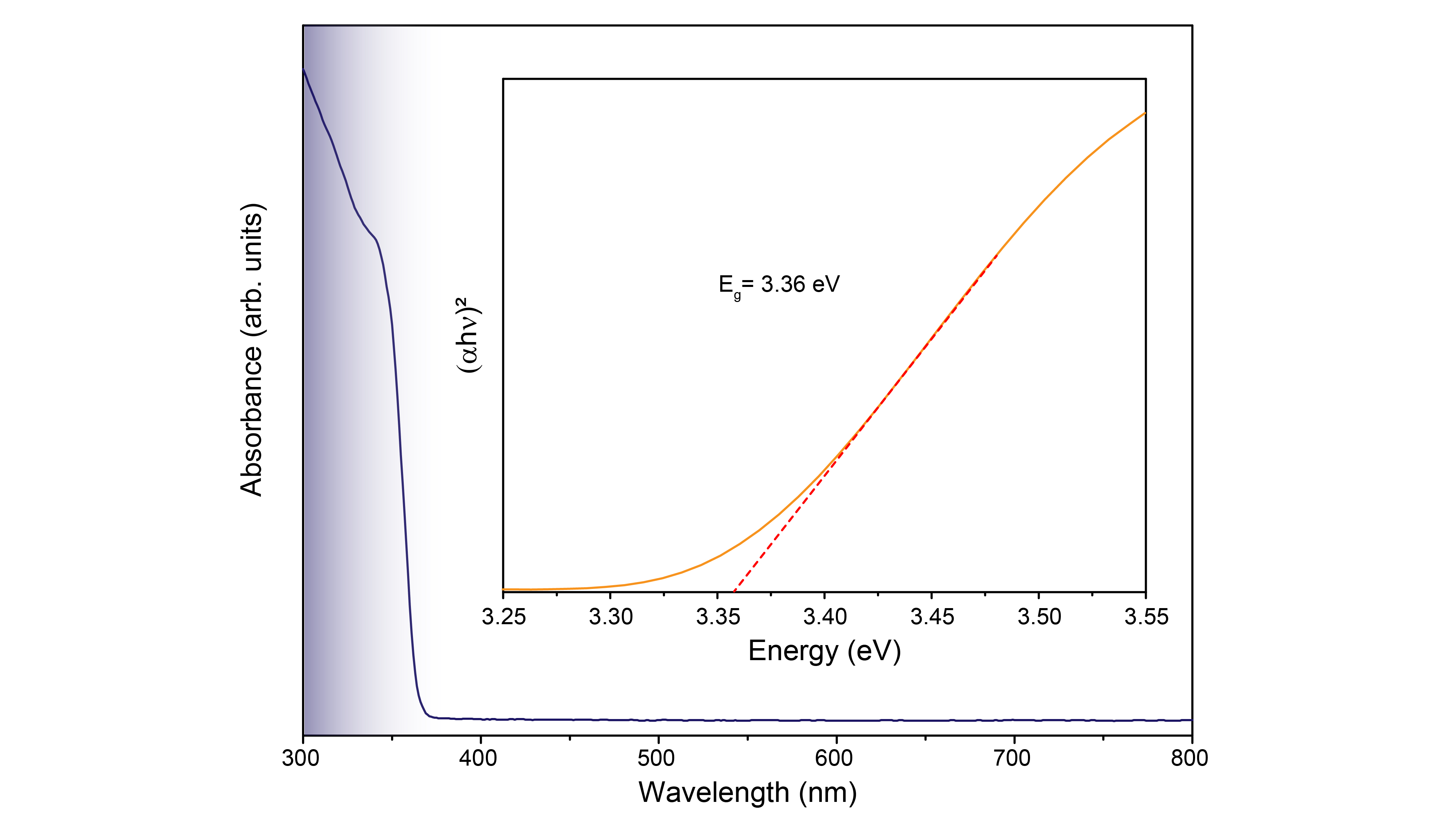


# Figure S3. Absorption spectrum for the ZnO QDs, the insert shows the band gap of the ZnO QDs.


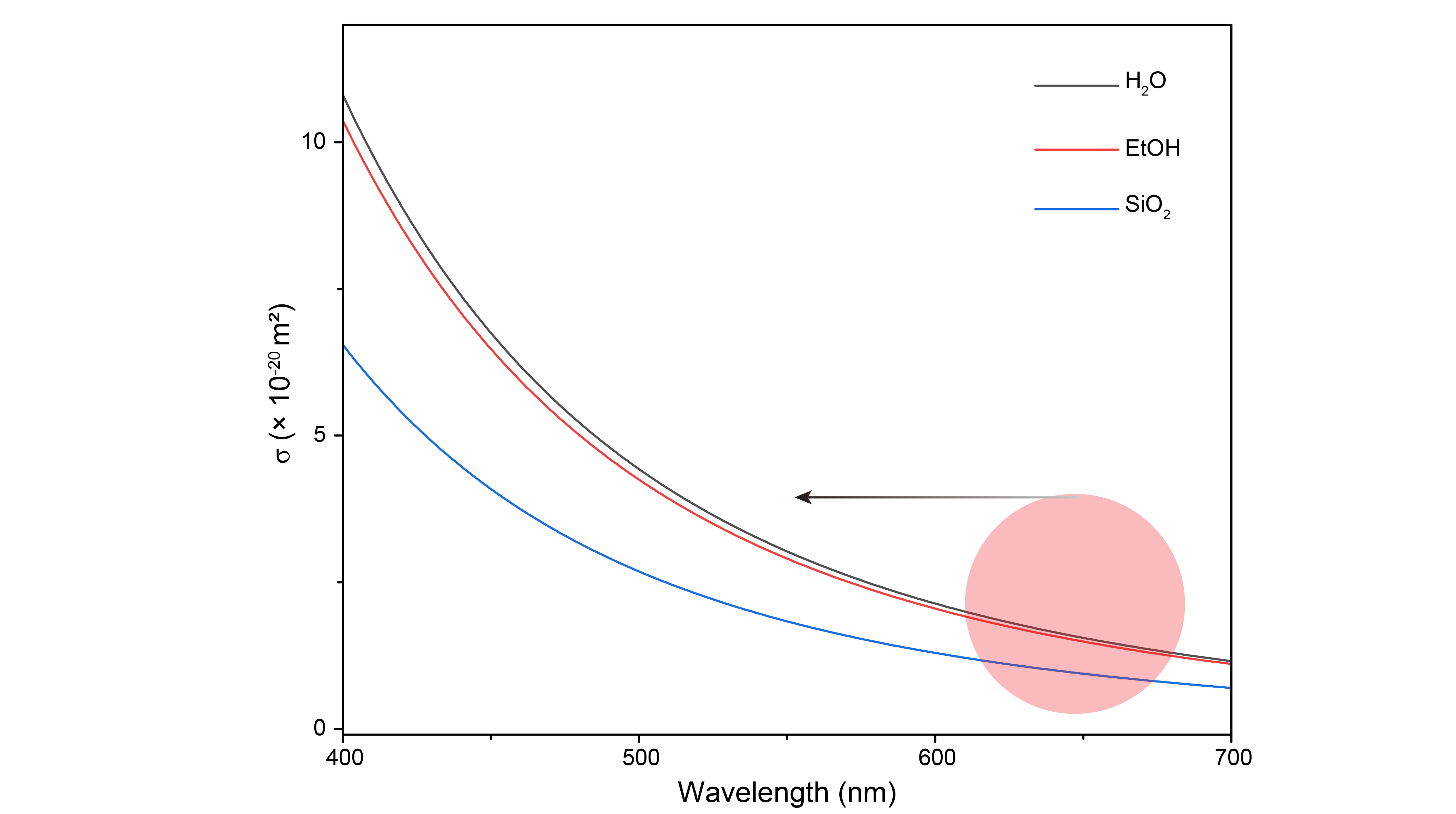


# Figure S4. Calculated Rayleigh scattering cross-section (σ) of the ZnO QDs versus wavelength, in water, ethyl alcohol and SiO_2_ matrix.


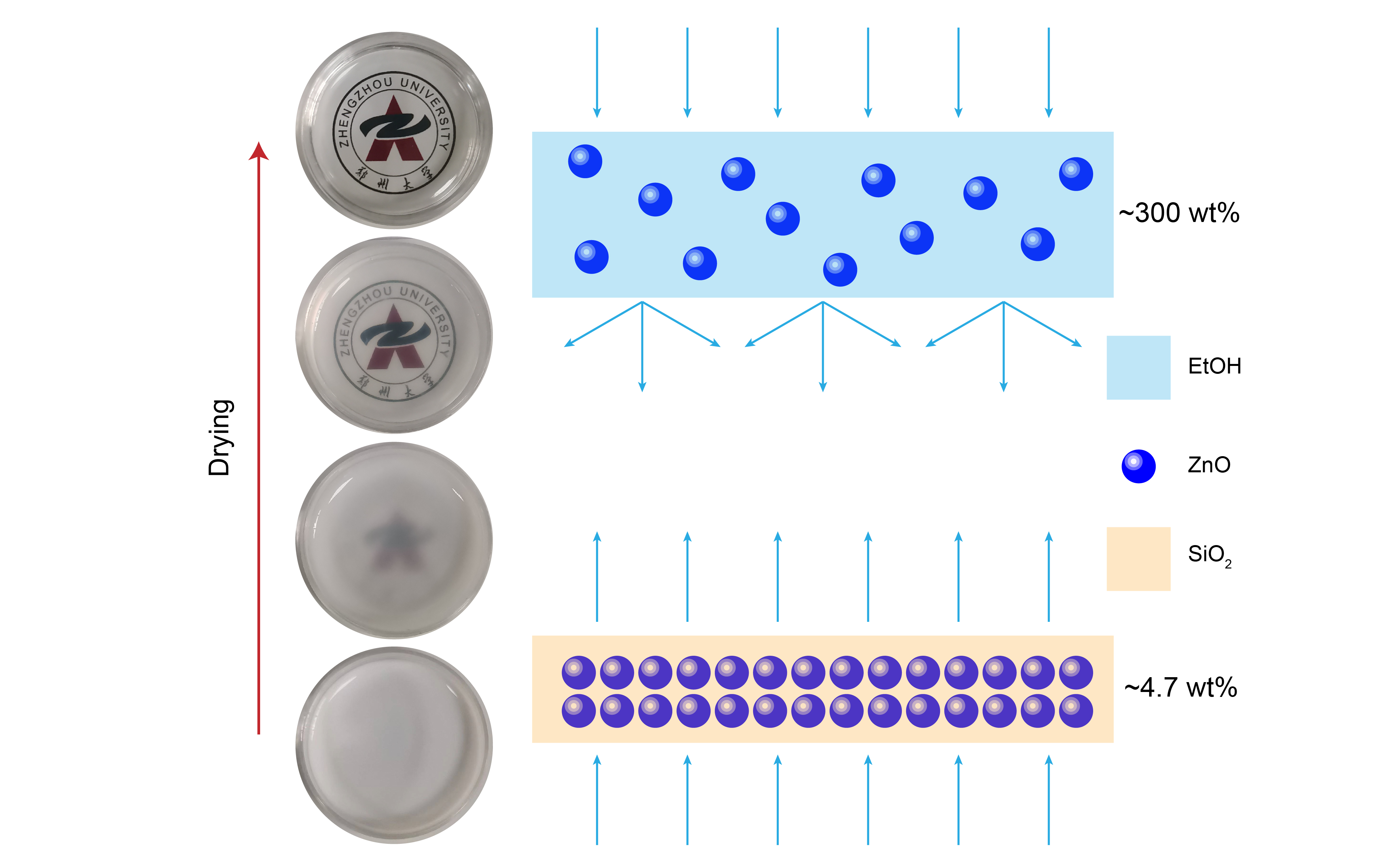


# Figure S5. Photographs of the scintillator from opaque-to-transparent (left), and the schematic diagram (right) of the varied scattering effect from two interfaces: EtOH/QDs and SiO_2_/ QDs.


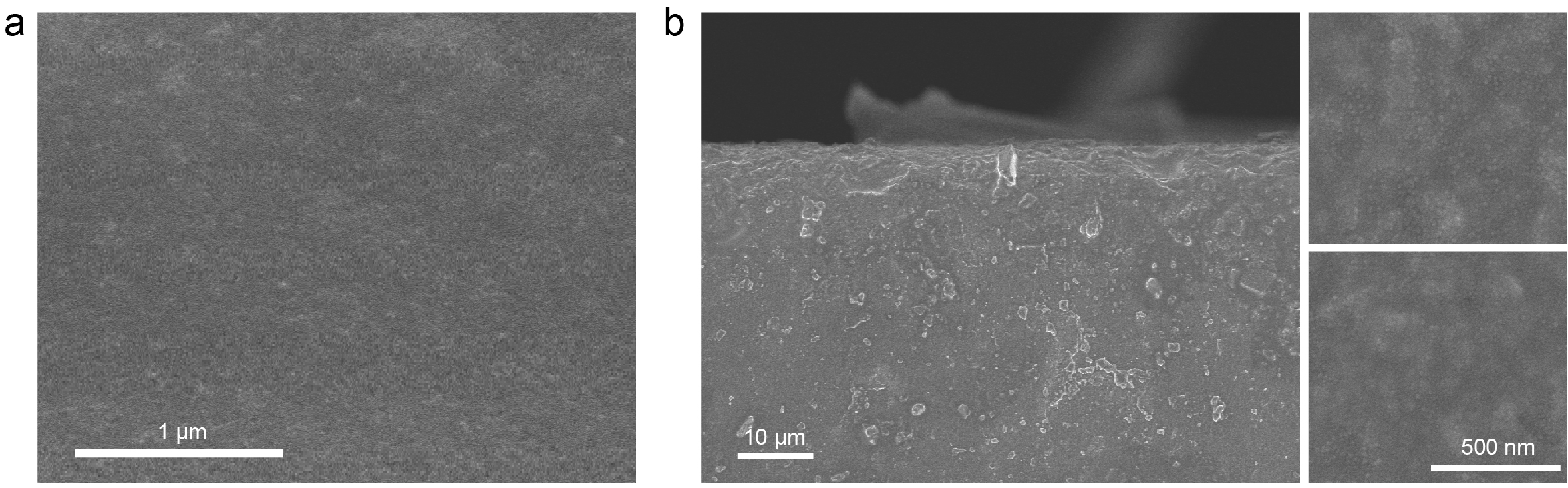


# Figure S6. (a) Top-view scanning electron microscopy (SEM) image of the ZnO QD vitreous scintillator. (b) The cross-section scanning electron microscopy (SEM) images, showing a flat surface and particle-packed interior.


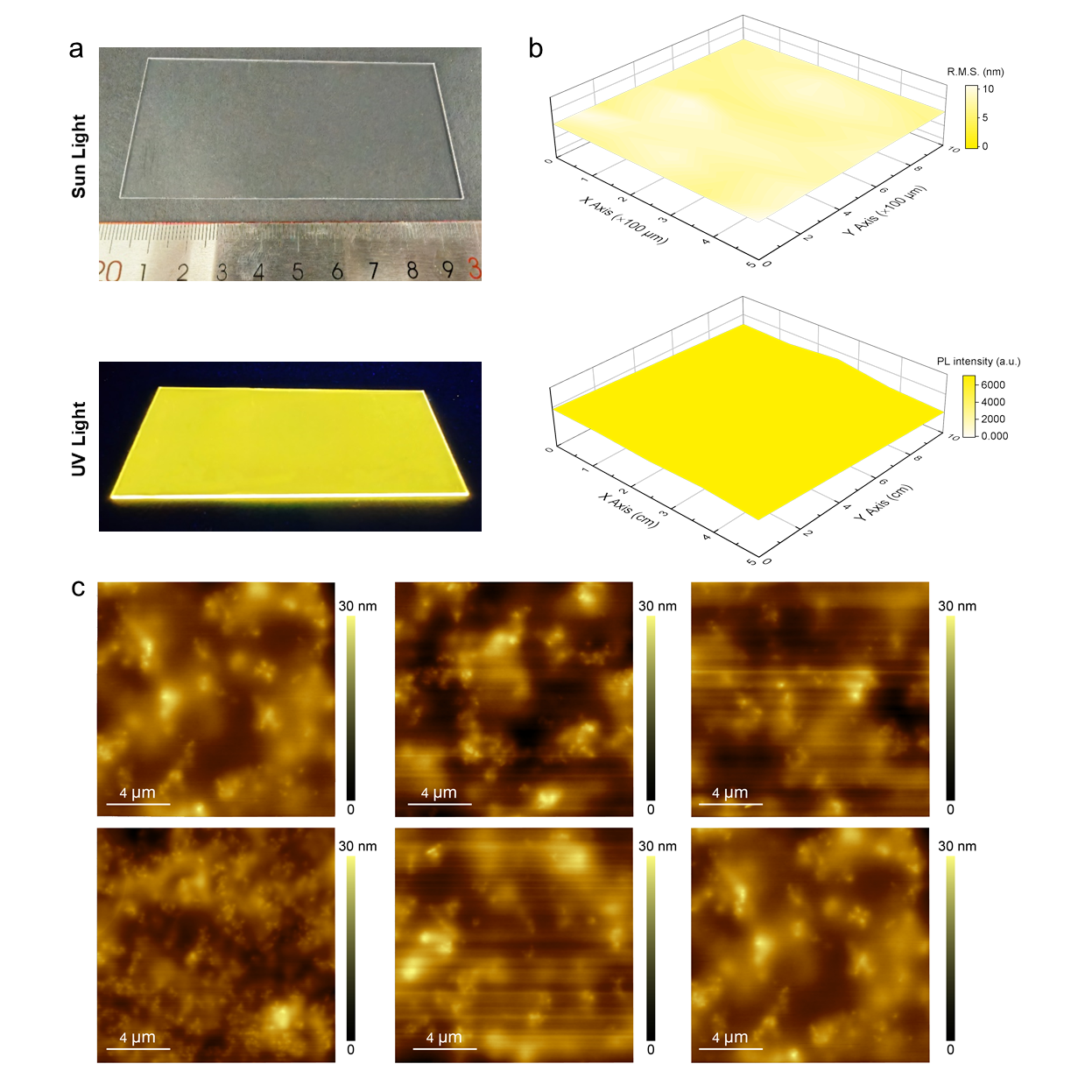


# Figure S7. (a) Photographs of 10.0 × 5.0 cm^2^ ZnO QD scintillator films under sun light and UV (365 nm) light irradiation. (b) AFM surface roughness (upper) and PL intensity (bottom) distribution of the ZnO QD scintillator films. (c) AFM images of six random regions of a ZnO QD scintillator films.


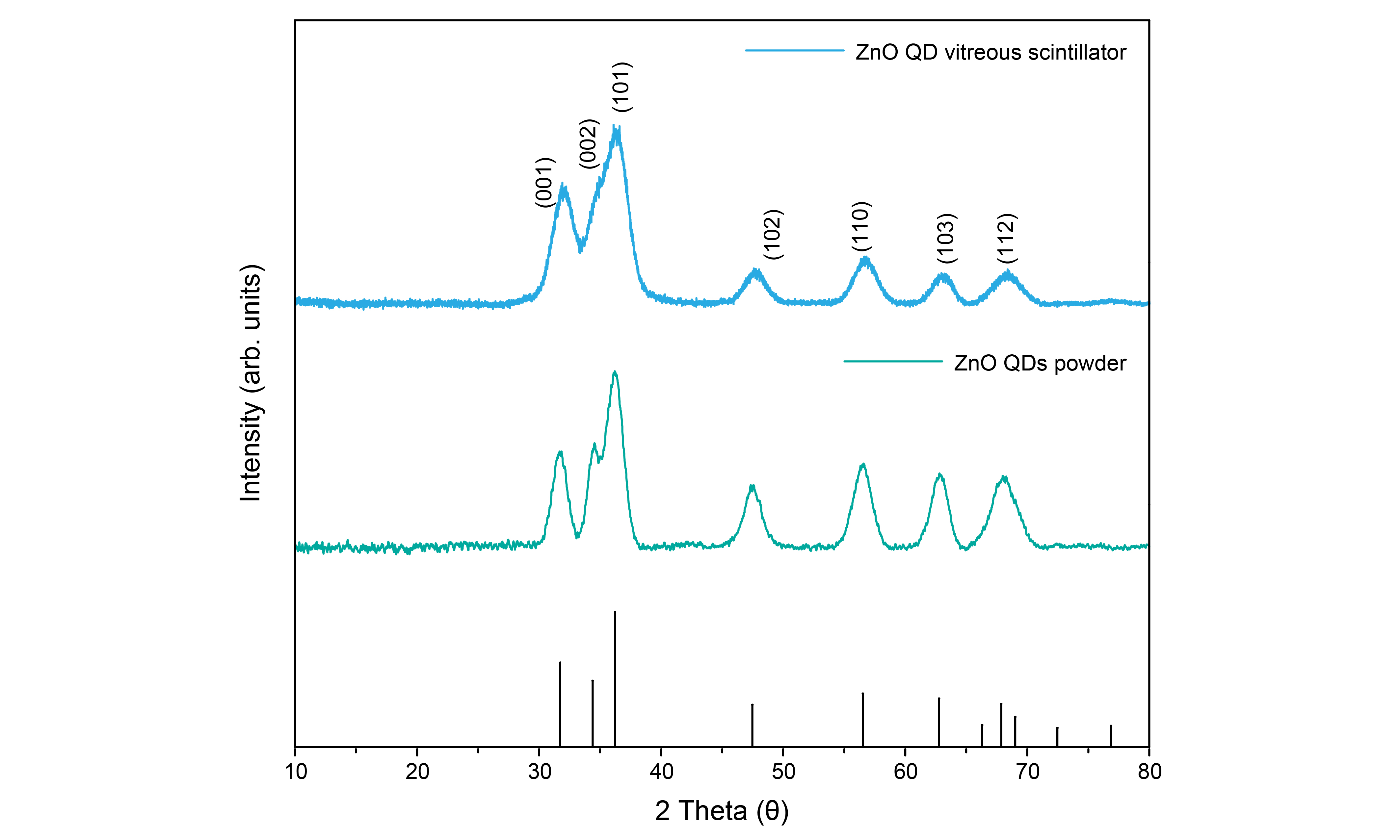


# Figure S8. XRD pattern of the ZnO QD powders and ZnO QD vitreous scintillator.


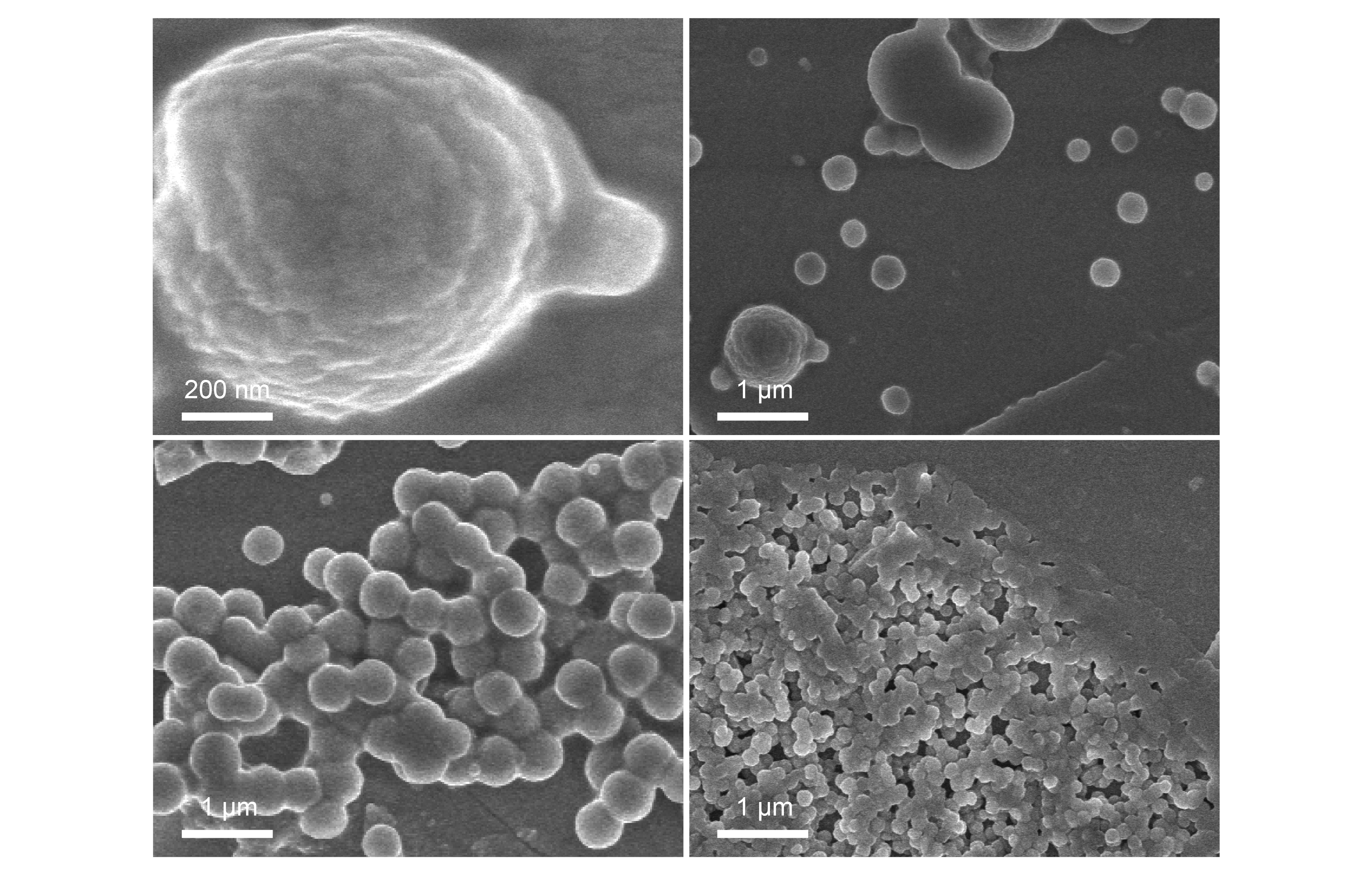


# Figure S9. SEM images of the ZnO QD vitreous scintillator captured at various formation processes.


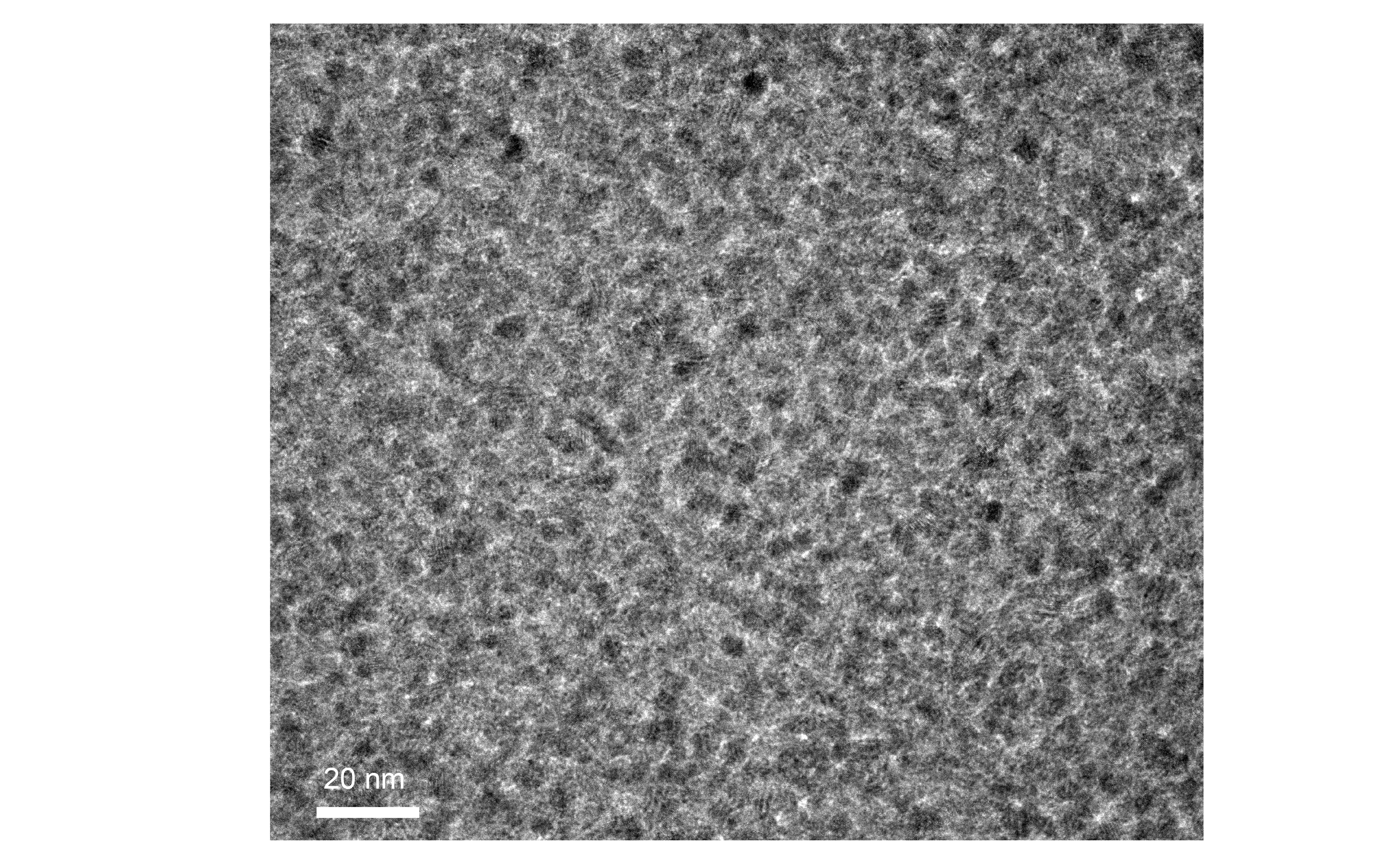


# Figure S10. TEM image of the ZnO QD vitreous scintillator.


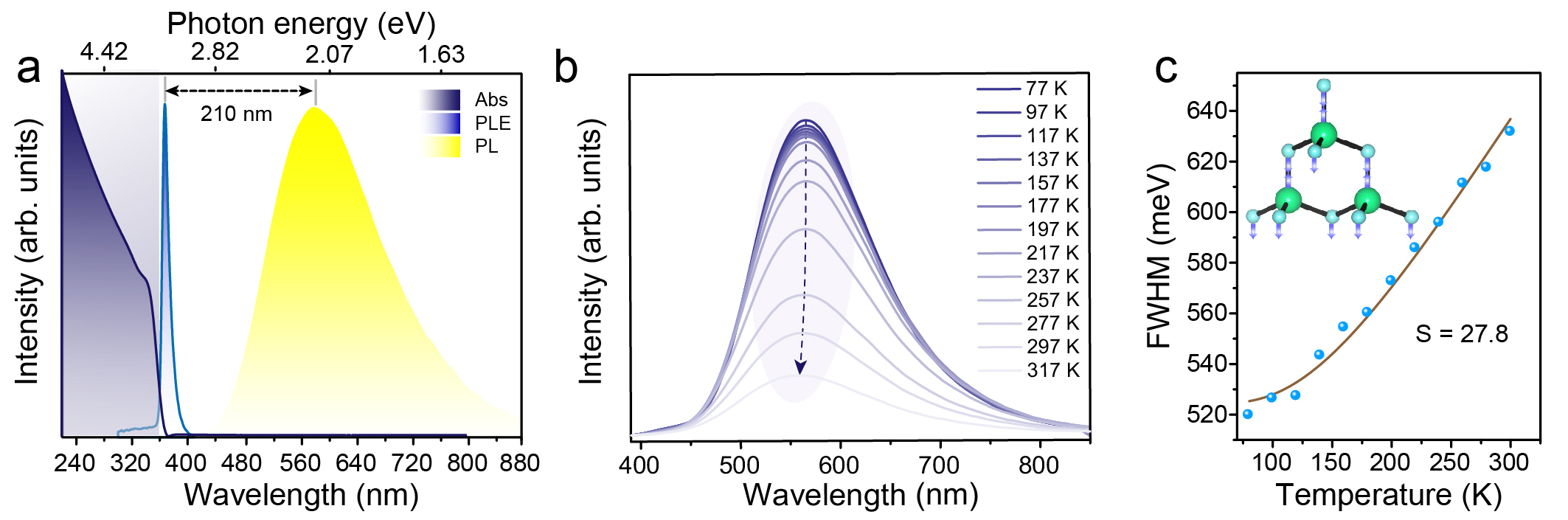


# Figure S11. (a), Absorbance, PL, and PL excitation spectra of ZnO QDs. (b) Temperature-dependent PL spectra of ZnO QDs measured from 77 to 317 K. (c) Temperature-dependent FWHM emission spectrum, and the corresponding fitting curve result (insert: schematic diagram of optical phonon vibration in ZnO QDs).


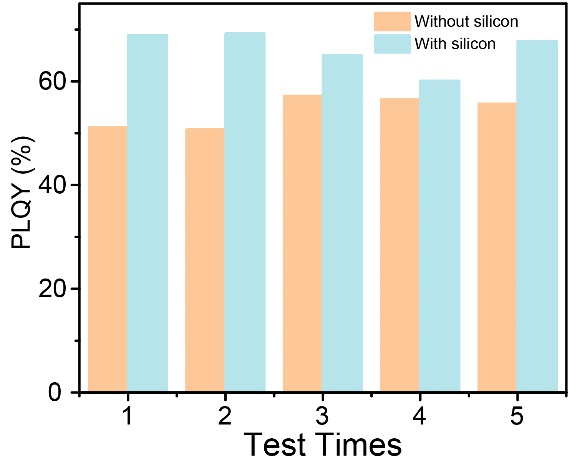


# Figure S12. The PL QY of ZnO QDs tested for five times before and after coating the silicon shell.


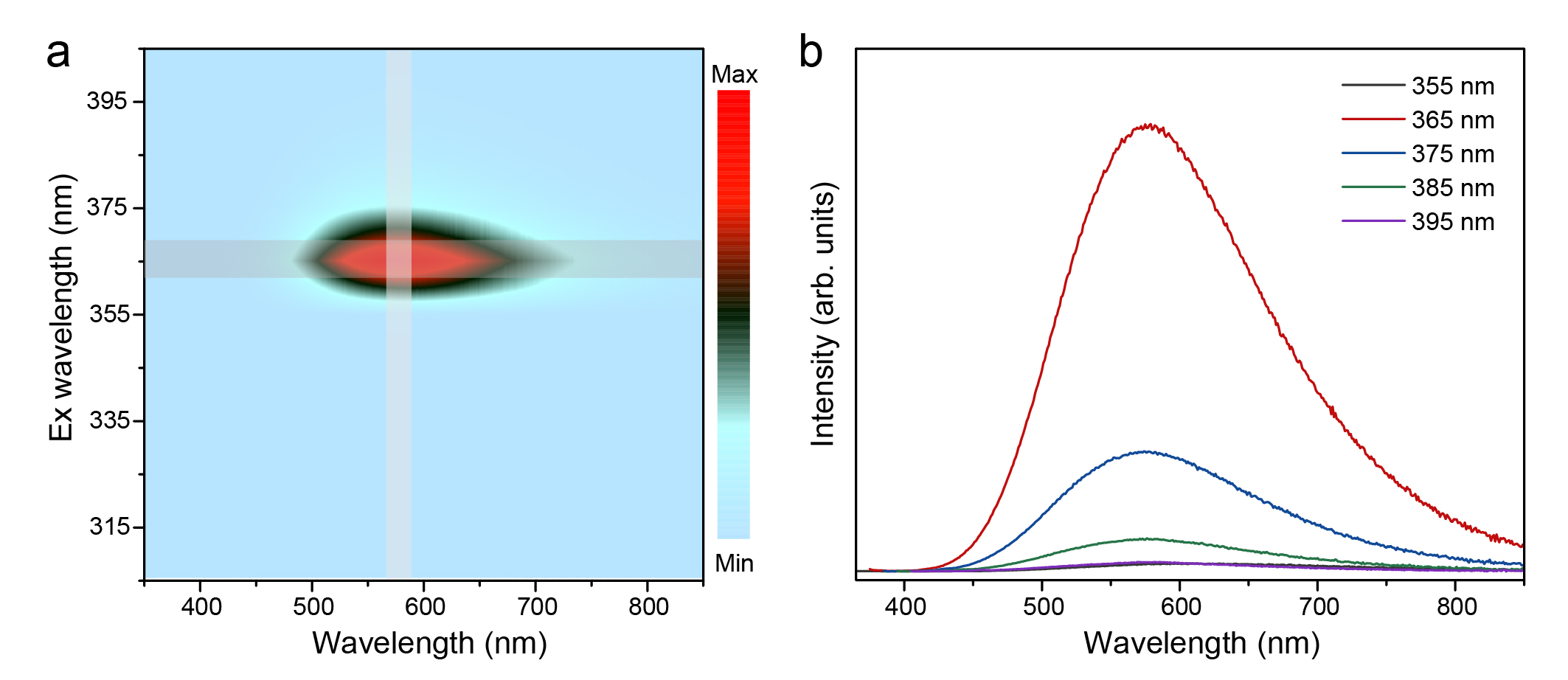


# Figure S13. (a) Excitation-Emission mapping of the ZnO QD vitreous scintillator. (b) The PL spectra of the ZnO QD vitreous scintillator under different excitation wavelengths.


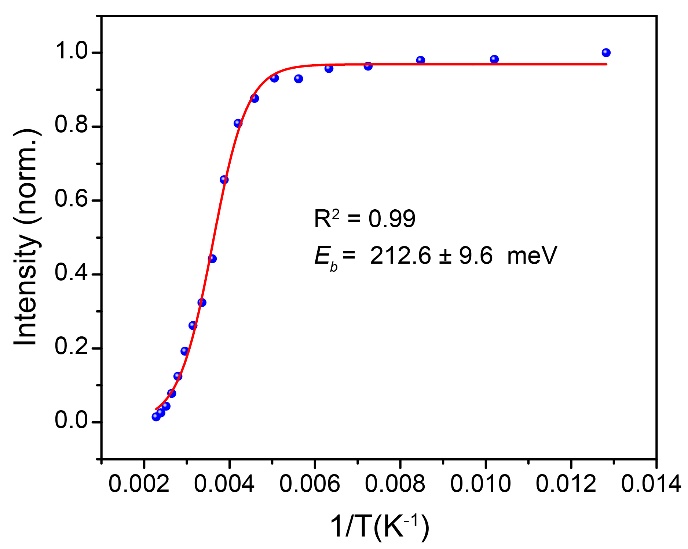


# Figure S14. Temperature-dependent integrated STE emission intensity of ZnO nanocrystals (218 K-418 K).


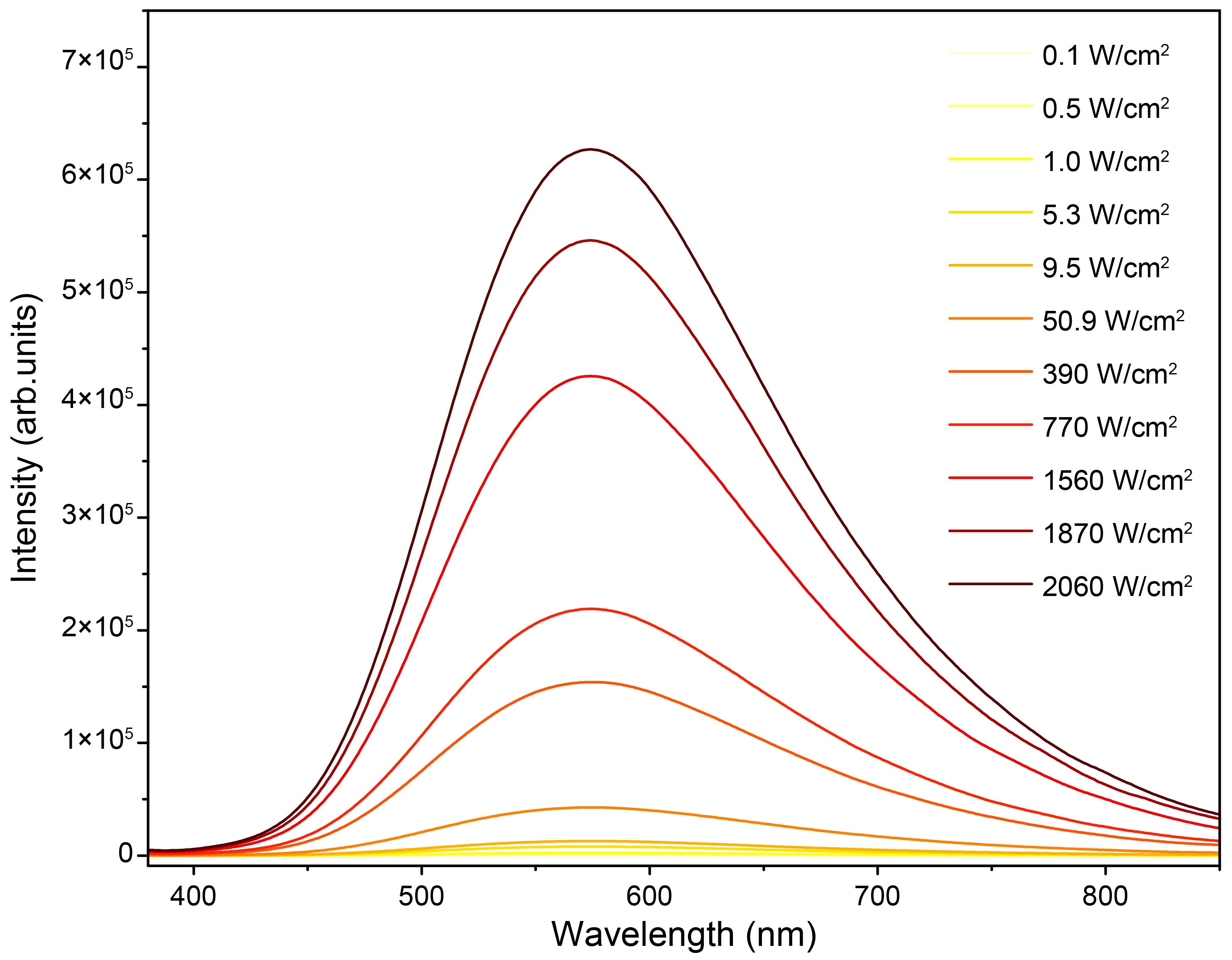


# Figure S15. The PL spectra of the ZnO QDs under excitation of laser with varying powers.


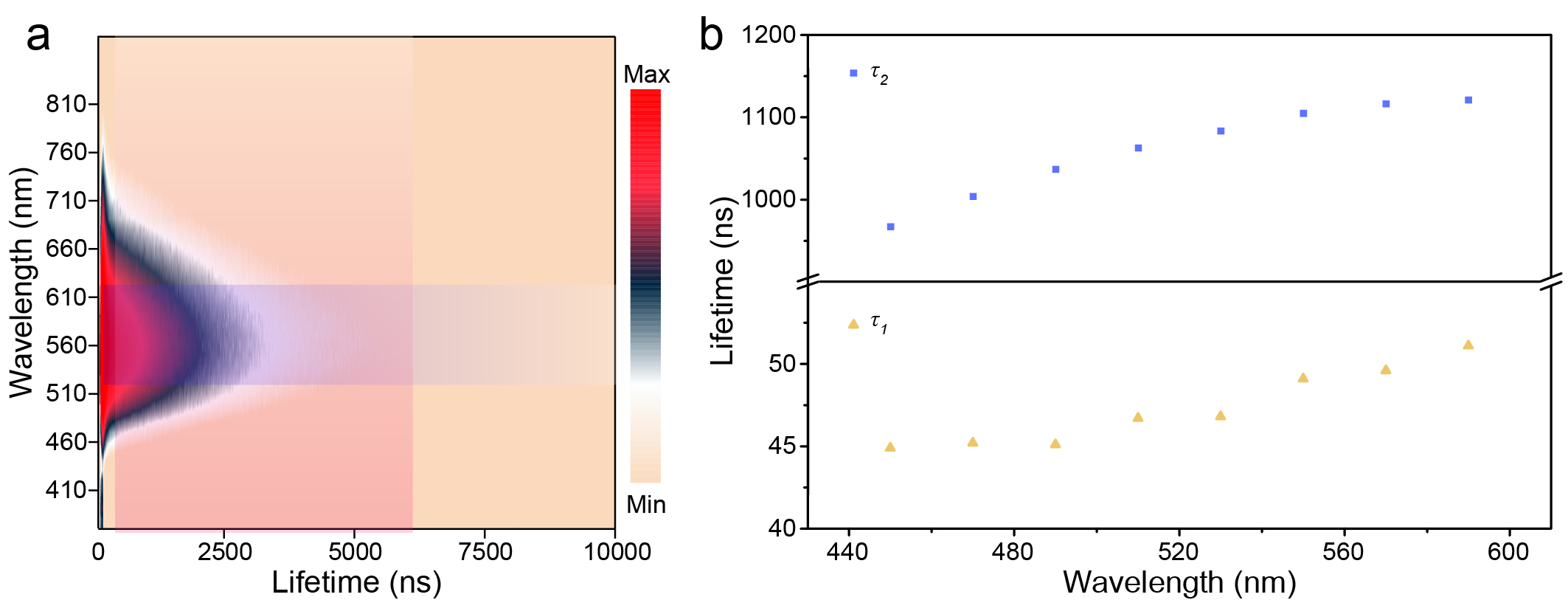


# Figure S16. (a) Contour plots of time-resolved emission spectra of the ZnO QDs. (b) The PL lifetime changes of the ZnO QDs at selected wavelength (450 nm-590 nm) under 340 nm excitation.


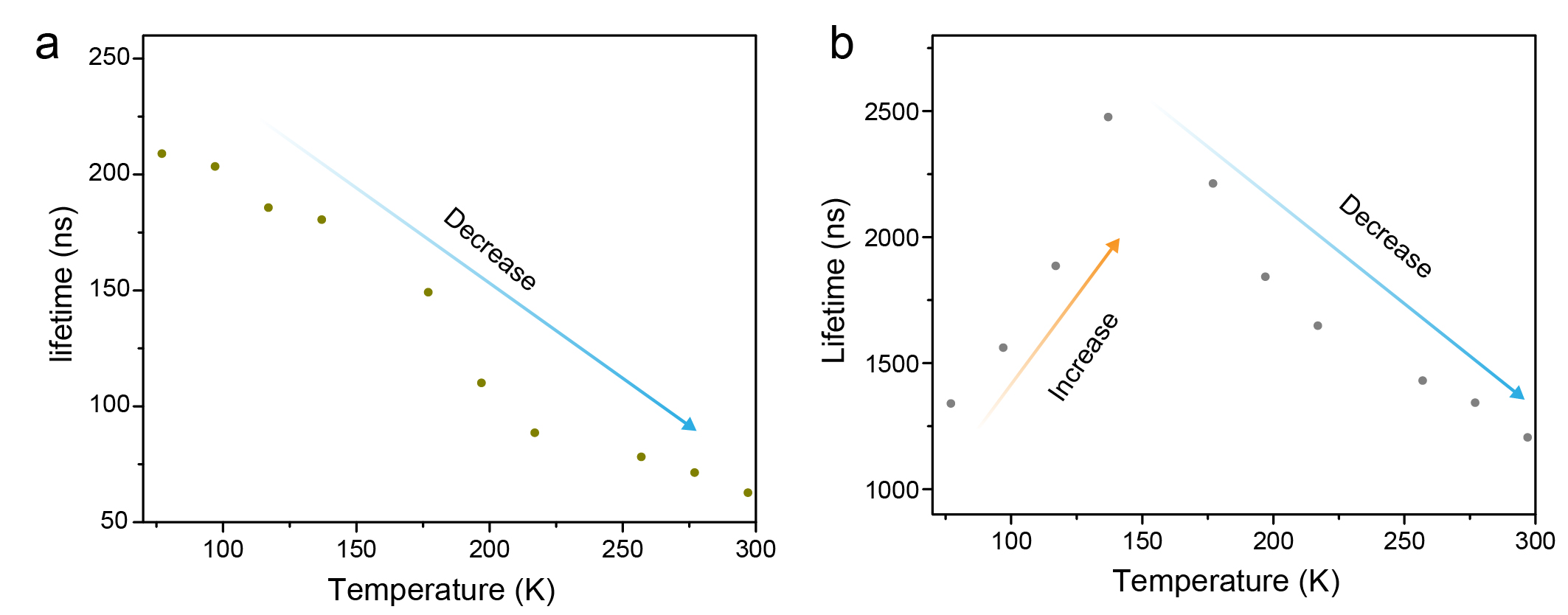


# Figure S17. (a) The temperature-dependent PL short lifetime and (b) long lifetime for the ZnO QDs.


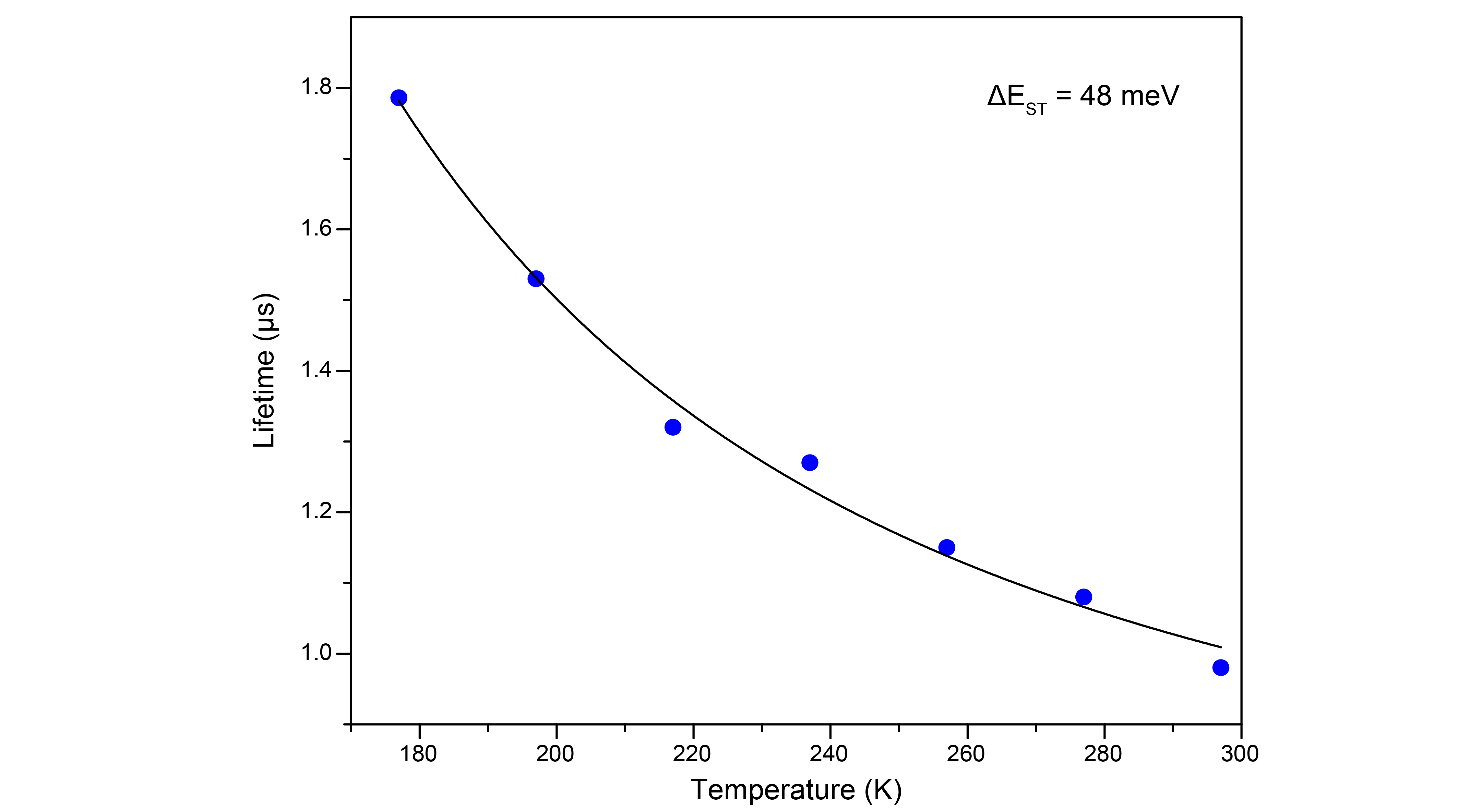


# Figure S18. Temperature-dependent average lifetimes of the ZnO QDs.


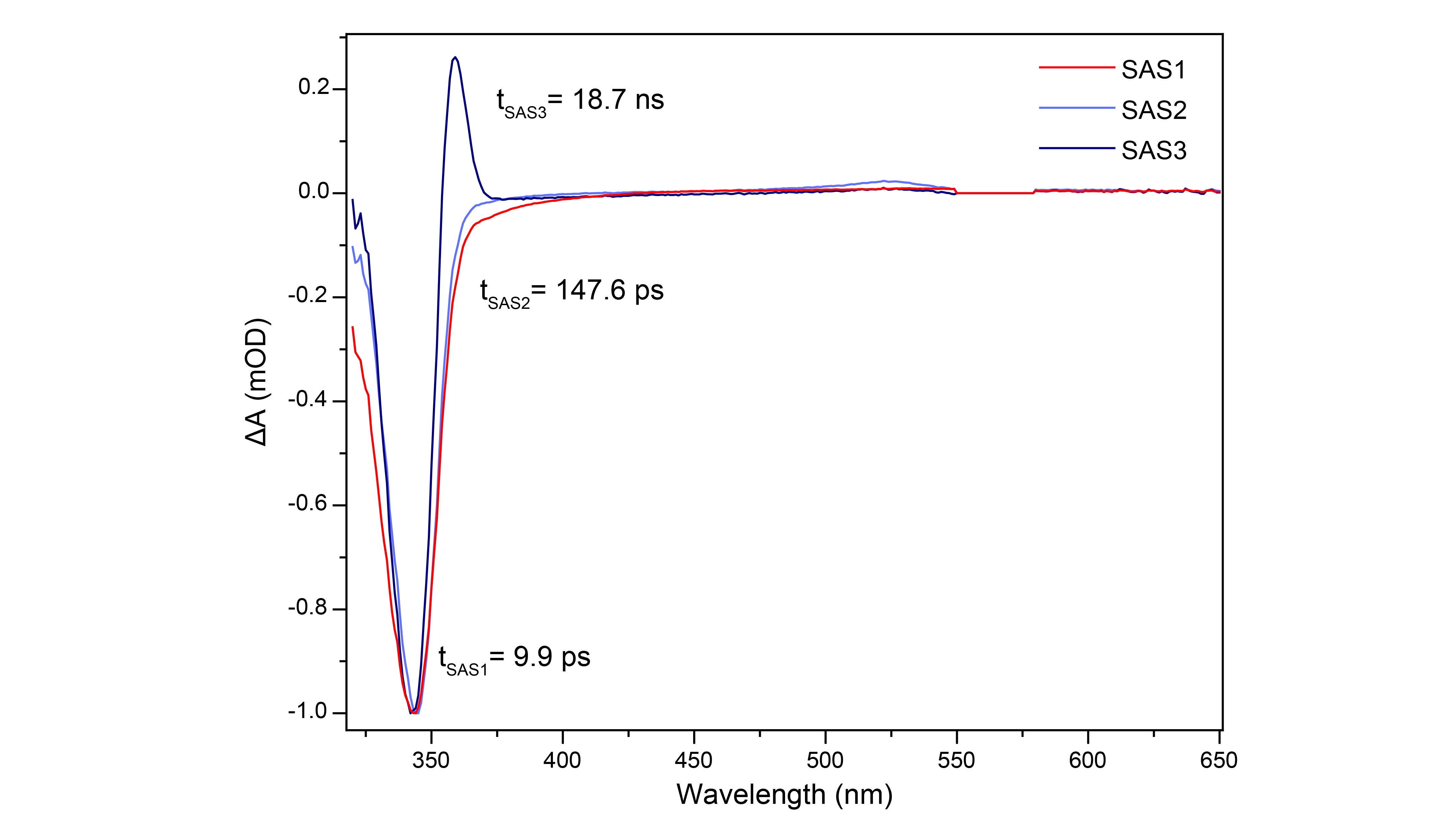


# Figure S19. Global analysis TA spectra of the ZnO QDs


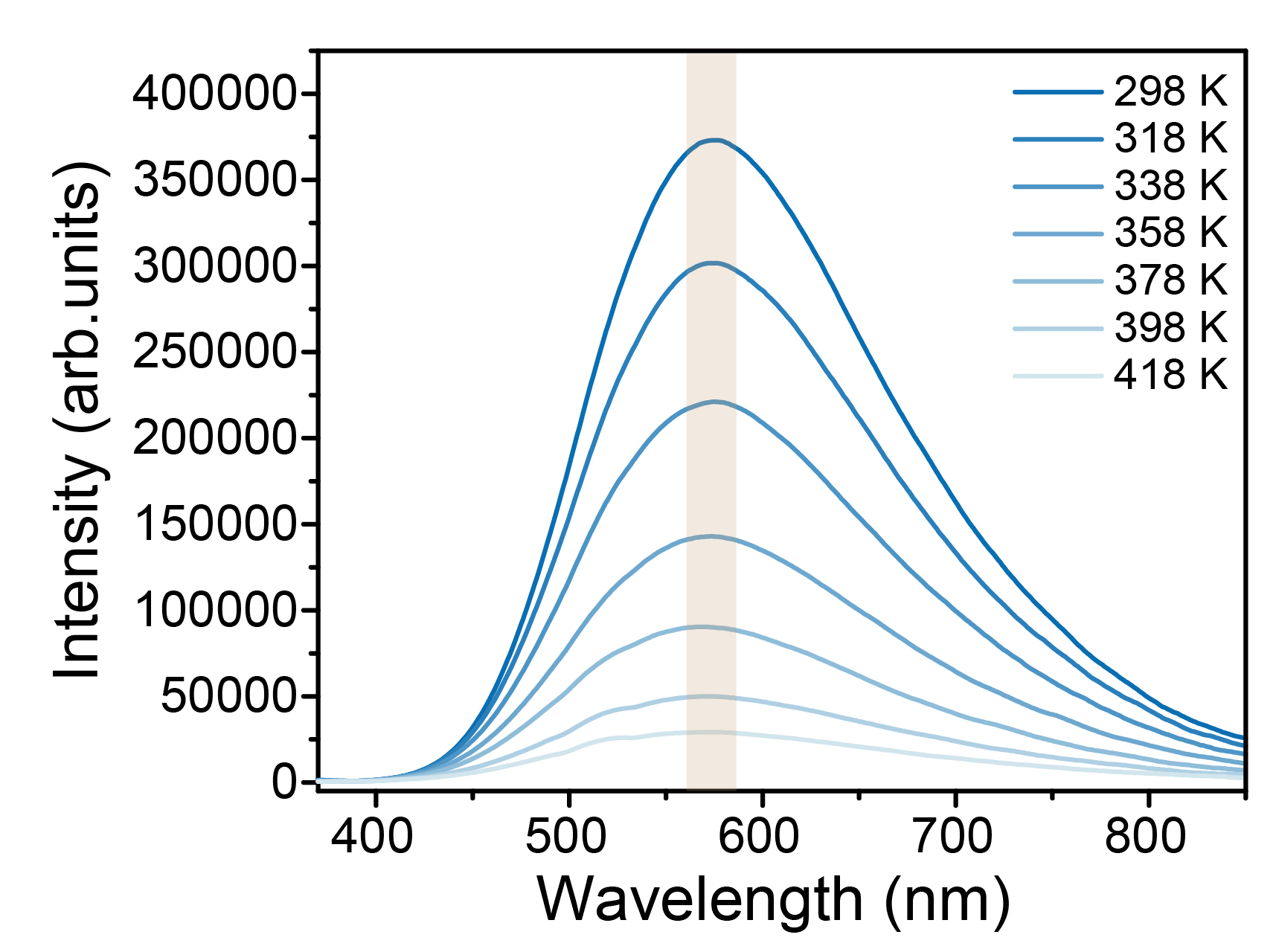


# Figure S20. Temperature-dependent PL spectra of ZnO QDs measured from 298 to 418 K.


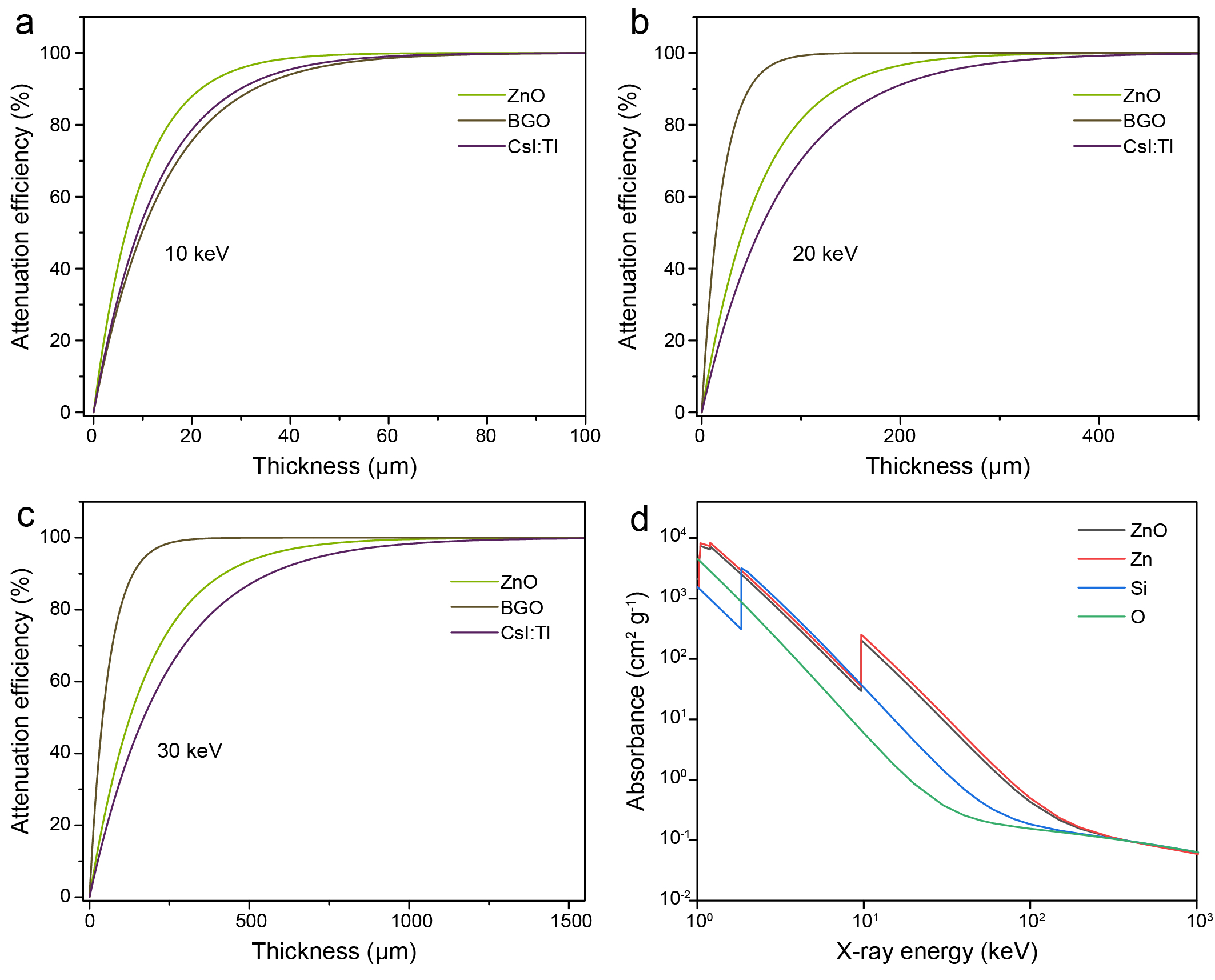


# Figure S21. (a-c) Calculated X-ray attenuation efficiencies of BGO, CsI:Tl and ZnO versus their thickness. (d) Absorption spectra of ZnO, Zn, Si and O as a function of X-ray energy. The attenuation coefficients were obtained from the photon cross-section database.


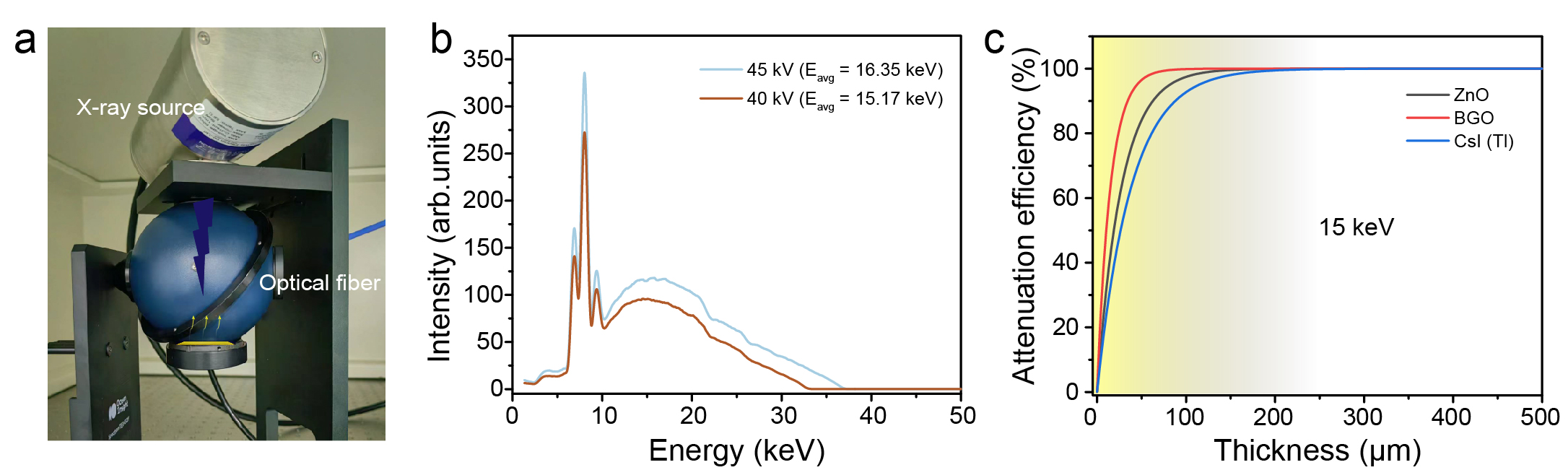


# Figure S22. (a) Photograph of the radioluminescence (RL) measurement system. (b) X-ray photon energy spectra generated by copper target at accelerating voltage of 40 kV and 45 kV. (c) X-ray attenuation efficiencies of the ZnO QD glassy scintillator, CsI: Tl and Bi_4_Ge_3_O_12_ (BGO) versus varied thicknesses at 15 keV.

The light yield was determined by Ocean optics, QE Pro spectrometer and optic-fiber coupled calibrated integrating sphere in system (Figure RS23a). The scintillators of appropriate thickness are selected to ensure that the absorption efficiency was carefully corrected at X-rays wavelength. Under X-rays excitation, the RL signal was collected by an integrating sphere and transmitted to the Ocean optics, QE Pro spectrometer through the optical fiber. The light yield (≈10000 photons/MeV) of the commercial scintillator BGO with a thickness of 1 mm is calibrated that was employed, and the light yield of the ZnO films (~200 μm) is calculated according to the RL spectra by using the following equation:

$$\frac{{LY}_{BGO}}{{LY}_{ZnO}}=\frac{\eta_{BG0}}{\eta_{ZnO}}\times\frac{\int I_{ZnO}\left( \lambda\right)S\left( \lambda\right)d\lambda/\int I_{ZnO}\left( \lambda\right)d\lambda}{\int I_{BGO}(\lambda)S\left( \lambda\right)d\lambda/\int I_{BGO}(\lambda)d\lambda}\times\frac{S_{BGO}}{S_{ZnO}}$$

Where *η* is the X-ray deposited energy percentage of scintillators, *I* is the RL intensity at different wavelength (λ), $S\left( \lambda\right)$is detection efficiency of the detector. *S* is the area exposed to X-ray of scintillators (*S_ZnO_*: π*56.25 mm^2^, *S_BGO_*: 10 mm * 10 mm), and the X-ray intensity is assumed to be uniform across the whole scintillator film. The measured *Vp* of X-ray tube was set at 40 kV and the average X-ray photon energy was calculated at 15.17 keV, as shown in Figure R23b. The *η*(BGO) and *η*(ZnO) was calculated at 100% according to the relationship between deposited energy percentage and thickness (Figure R23c). Based on these, the Light yield of ZnO films was calculated to be about 16296 ± 1754 photons MeV^-1^ as shown in Figure R18d. The measurement system was cross-checked with another commercial scintillators of CsI:TI and LYSO, obtaining a light yield of 53000 photons MeV^-1^ and 29200 photons MeV^-1^, which matches their datasheet value (~54000 photons MeV^-1^) and (33000 photons MeV^-1^) proves the validity of the measurement method.


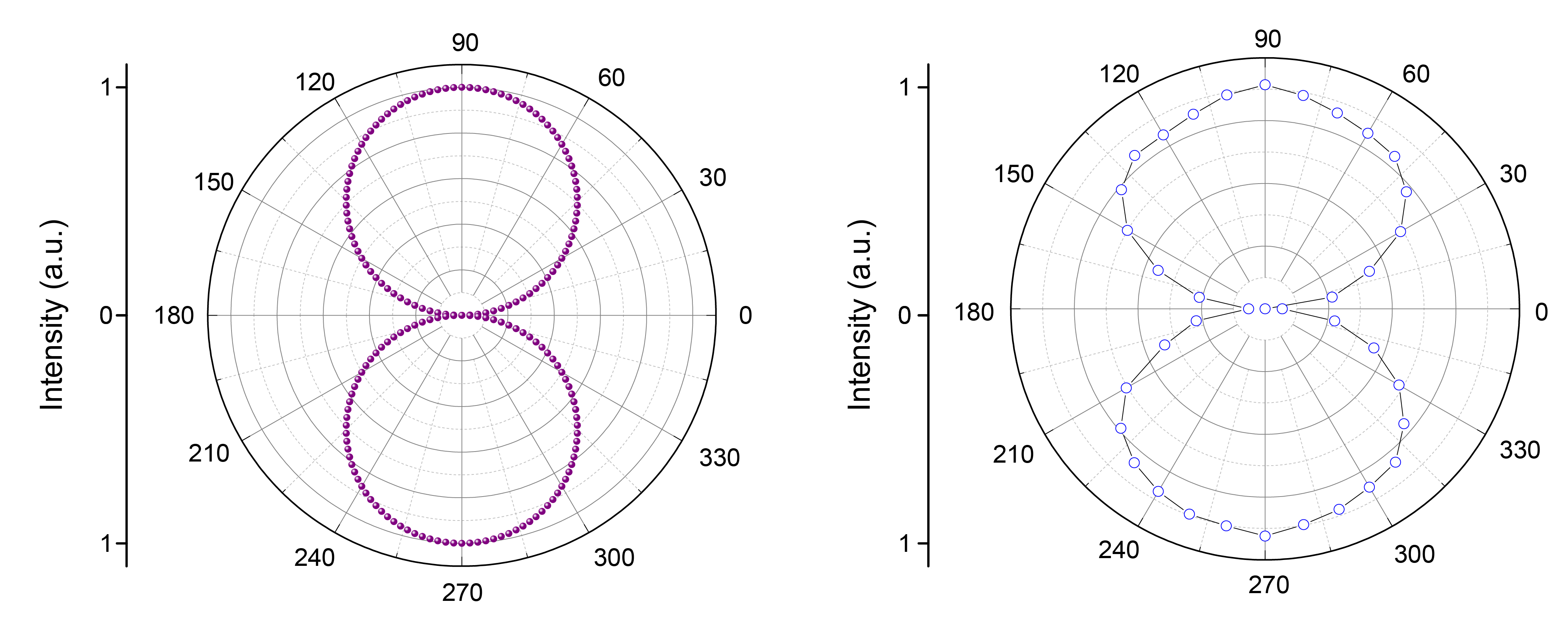


# Figure S23. Simulated light intensity in different directions at the same dose rate (left) and measured light intensity in different directions (right).


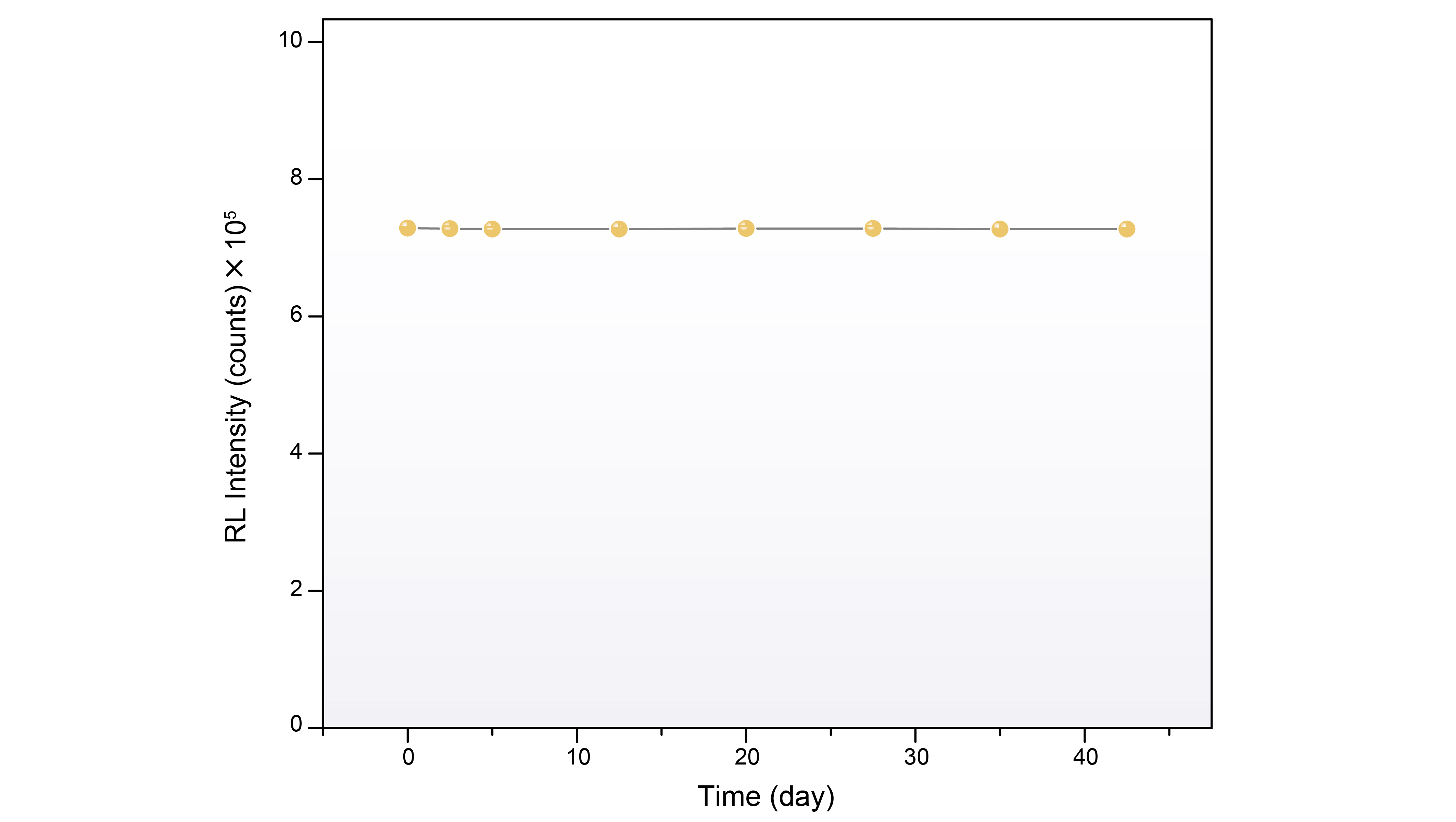


# Figure S24. RL stability of the ZnO QD vitreous scintillator under X-ray irradiation versus times.


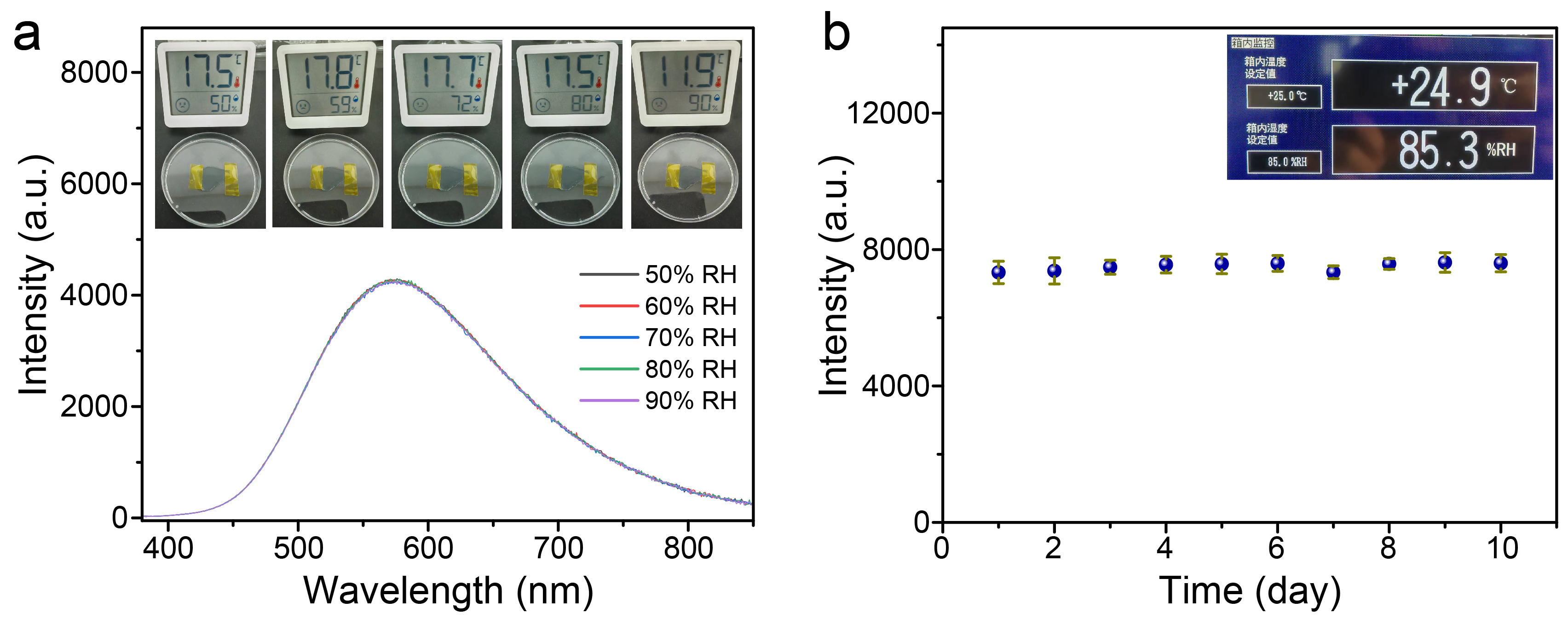


# Figure S25. (a) The PL spectra of ZnO scintillator under different relative humidity from 50% to 90%. The inserts are the photographs of ZnO scintillator under day light. (b) The curve of the emission intensity of ZnO scintillator versus time with a relative humidity of 85%.


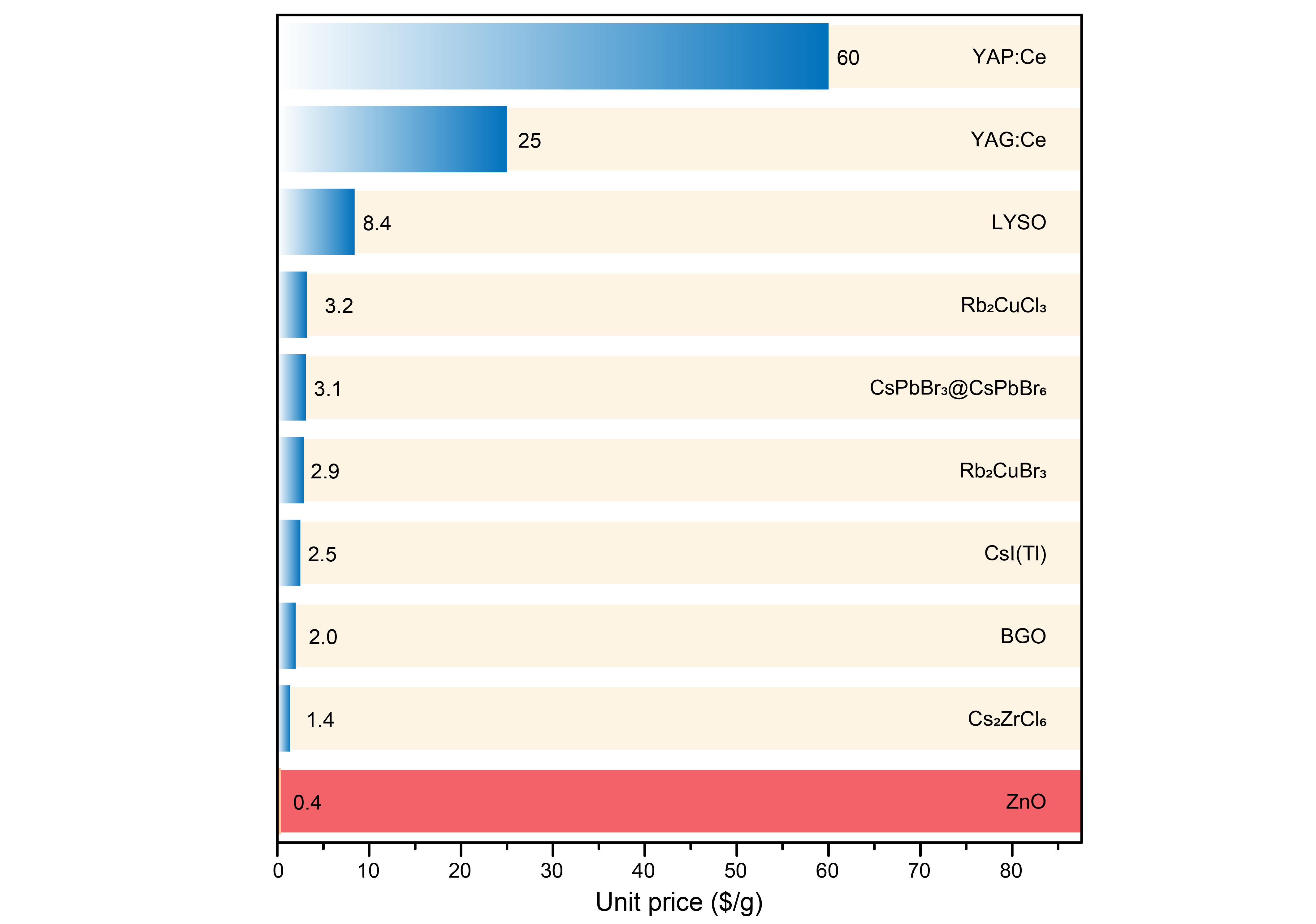


# Figure S26. Cost/price comparison of the ZnO QD and other scintillators.


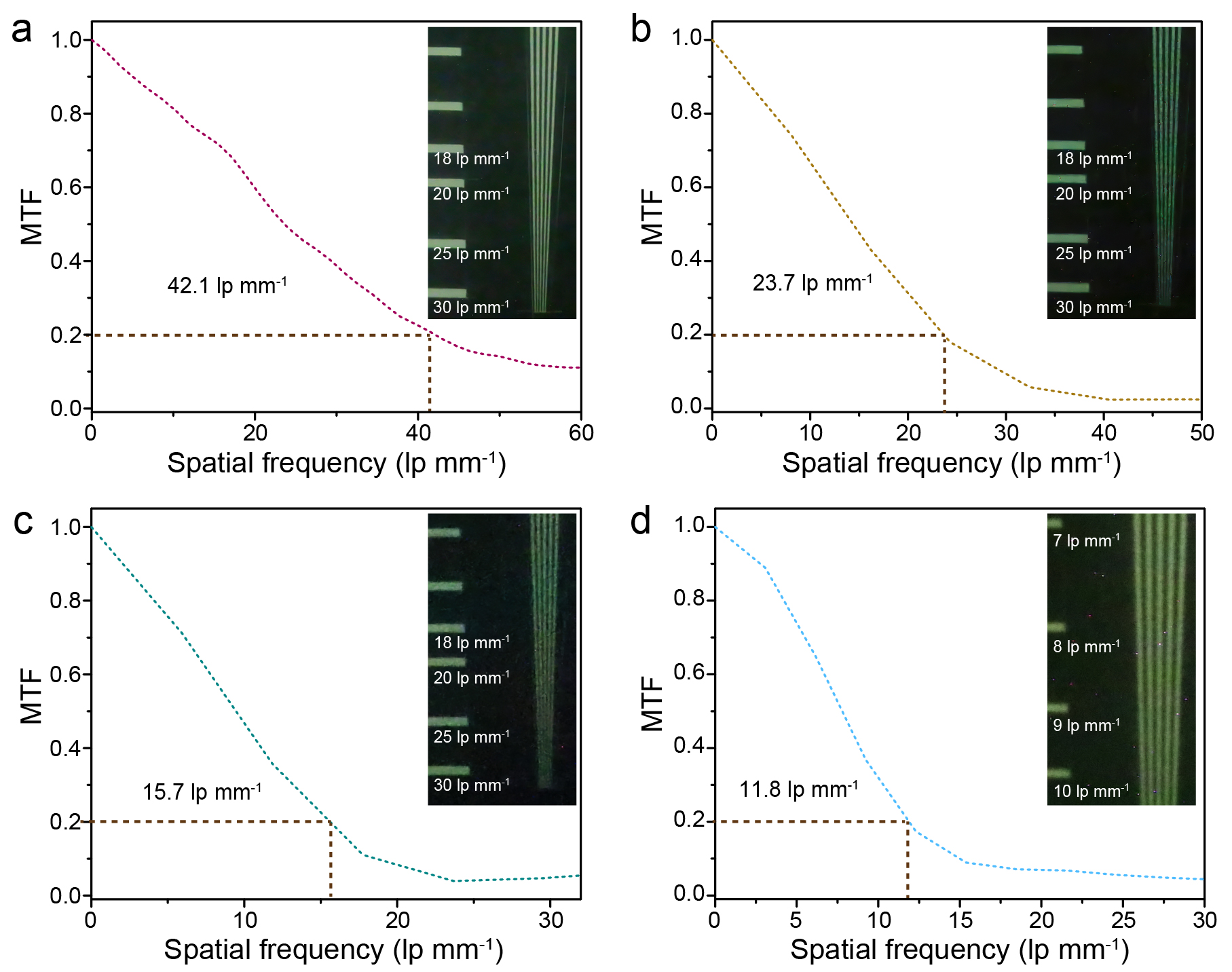


# Figure S27. The spatial resolution of the ZnO QD scintillator with a thickness of 0.1 mm (a), 0.2 mm (b), 0.5 mm (c), 1 mm (d). Insert: photographs of the test chip and the corresponding X-ray image.


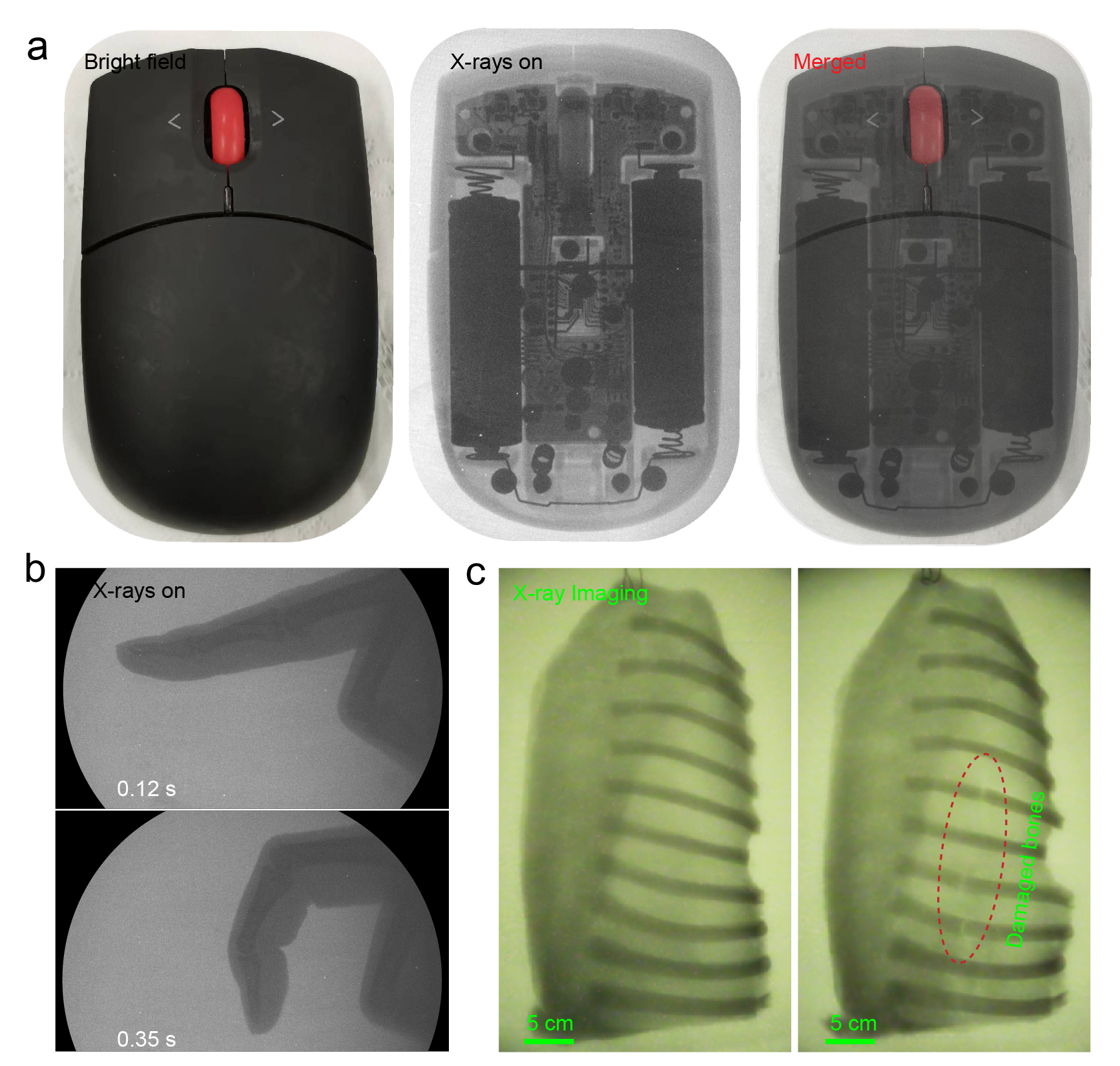


# Figure S28. (a) Digital photograph of a computer mouse and corresponding X-ray images obtained using ZnO QD vitreous scintillator (50 kV and 258 μGy s^−1^ exposure for 200 ms). (b) Real-time X-ray images of finger bending. (c)X-ray imaging of pork rib using the large area ZnO QD scintillator.
